# COVID-19 collateral damage—psychological burden and behavioural changes among older adults during the first outbreak in Stockholm, Sweden: a cross-sectional study

**DOI:** 10.1136/bmjopen-2021-058422

**Published:** 2022-01-07

**Authors:** Giorgi Beridze, Federico Triolo, Giulia Grande, Laura Fratiglioni, Amaia Calderón-Larrañaga

**Affiliations:** 1 Aging Research Center, Department of Neurobiology, Care Sciences and Society, Karolinska Institutet, Solna, Sweden; 2 Stockholm Gerontology Research Center, Stockholm, Sweden

**Keywords:** COVID-19, epidemiology, geriatric medicine

## Abstract

**Objectives:**

To explore the indirect negative effects of COVID-19 restrictions (collateral damage) on the lives and health of older adults living in central Stockholm, and to characterise the sociodemographic profile of those with the highest susceptibility to this damage.

**Design:**

Cross-sectional study.

**Setting:**

District of Kungsholmen in Stockholm, Sweden.

**Participants:**

Older adults aged 68 years and above (n=1231) who participated in the ad hoc COVID-19-related phone questionnaire administered by trained staff between May and June 2020 and who had previously attended the regular follow-up assessment of the Swedish National study on Aging and Care in Kungsholmen (SNAC-K) during 2016–2019.

**Primary and secondary outcome measures:**

Three dimensions of collateral damage: psychological burden (feelings of worry, stress and loneliness), reductions in social and physical activities, and reductions in medical and social care use since the beginning of the pandemic. Logistic regression models were used to test the association between age, sex, education and living arrangement, and the risk of collateral damage.

**Results:**

Vast majority of participants adhered to the national public health recommendations, with over three-quarters practising self-isolation (n=928). Half of the sample reported psychological burden, 55.3% reported reductions in social or physical activity, and 11.3% reported decreased medical or social care use. Over three quarters of participants (77.8%) were affected by at least one of the three collateral damage dimensions. Female sex was the strongest sociodemographic predictor of both individual and co-occurring dimensions of collateral damage.

**Conclusions:**

COVID-19 and its restrictions during the first half of 2020 had a negative effect on the health and lives of a majority of the elderly living in central Stockholm. Women were at a higher risk of these negative consequences. We emphasise the need for predefined, evidence-based interventions to support those who are most susceptible to these consequences, both during the pandemic and once the outbreak is overcome.

**Strengths and limitations of this study**

- This study uses a large sample of older adults from a well-characterised population-based study (Swedish National study on Aging and Care in Kungsholmen, SNAC-K).
- Several dimensions of the indirect negative effects (collateral damage) of the COVID-19 pandemic are explored in this study.
- As Sweden’s response to COVID-19 differed from most countries, this study provides a unique opportunity for comparison with other settings.
- The cross-sectional design of this study does not allow to establish temporality between the onset of the pandemic and studied outcomes.
- The results of this study may not be generalisable to the entire elderly population in Sweden as participants are from an urban, affluent area in Stockholm.

## Introduction

The outbreak caused by the novel coronavirus, SARS-CoV-2, was declared a global pandemic by the WHO on 11 March 2020, coinciding with the date of the first confirmed death in Sweden. Early on, it was identified that older adults were at a significantly higher risk of mortality from COVID-19. Indeed, as of 18 January 2021, 91% of deaths attributed to COVID-19 in Sweden had happened in those aged 70 years and above.^1^ Later on, additional prognostic factors were identified, including male sex, socioeconomic disadvantage, the presence of comorbidities and frailty.^2–5^

In response to the pandemic, most countries have implemented strict measures to help curb the spread of the virus and reduce mortality. Sweden’s response to COVID-19 differed from most countries by not implementing strict lockdowns and restrictions, but instead relying on high voluntary adherence to the recommendations proposed by the Public Health Agency. The general recommendations included keeping good hand hygiene, practising social distancing and avoiding contact if having any symptoms.^6^ On top of these, the specific recommendations for the elderly were to stay at home, avoid social gatherings and public transportation, but to remain physically active outdoors in a safe manner.^6^

The importance of looking beyond mortality and morbidity when assessing national response strategies for COVID-19 has been raised.^7^ Stay-at-home orders and lack of contact with loved ones put the elderly at risk of loneliness and social isolation, which, in turn, are known to have negative effects, particularly in old age.^8^ Reduced physical activity and sedentarism have detrimental effects on physical and mental health.^9 10^ Additionally, due to the overburdening of healthcare services and reduced access to medical, social and informal care, new conditions may not be timely diagnosed, and existing health conditions may be exacerbated. These consequences can be considered indirect negative effects (ie, collateral damage) of COVID-19 restrictions.

While several studies have examined the distribution of COVID-19 mortality among Swedish older adults by sex, socioeconomic and household factors,^11–13^ little is known on the collateral damage of public health restrictions. To the best of our knowledge, only one study has examined the mental health consequences of the Swedish strategy on the elderly,^14^ and no study has looked into other dimensions such as psychological well-being and/or behavioural changes. Thus, the aims of this study were to explore different dimensions of the collateral damage linked to COVID-19 during the first outbreak in an older population of central Stockholm, as well as to characterise the sociodemographic profile of those with the highest susceptibility to this damage.

## Methods

### Study population

Study population consisted of 1231 older adults aged between 68 and 103 years, participating in the Swedish National study on Aging and Care in Kungsholmen (SNAC-K). SNAC-K is a longitudinal study including a random sample of older adults aged 60 years and above living in the Kungsholmen area of Stockholm, Sweden. All SNAC-K participants who had participated in the regular follow-up assessment during 2016–2019 were invited to participate in a telephone interview aimed at monitoring preventive behaviours and the direct and indirect health consequences of the COVID-19 pandemic. Most telephone interviews (95%) were conducted between May and June 2020 by trained SNAC-K staff, following a questionnaire that was developed ad hoc by the SNAC-K data collection team as well as by experts in geriatric medicine, neurology and public health. The questionnaire comprised a selection of items from the original SNAC-K assessments and from the WHO Europe survey tool to monitor the public’s risk perceptions, behaviours, trust and knowledge concerning the pandemic outbreak response.^15^ The interview was preceded by a brief explanation whereby participants were told that all questions referred specifically to the pandemic context. Exclusion criteria included living in care and nursing homes, known diagnosis of dementia, and very impaired hearing. The response rate was 91.9%. Subjects who refused to participate or could not be contacted had similar age and educational attainment to those who participated, but were more likely to be male (45.4% vs 35.7%, p=0.044).

### Collateral damage

In this study, we examined three dimensions of collateral damage: psychological burden and two aspects related to behavioural changes, that is, reductions in social and physical activities, and in medical and social care use. All questions explicitly asked participants about changes since the beginning of the pandemic. Psychological burden was assessed with variables related to worrying about being affected by COVID-19 (very/extremely vs not at all/somewhat/moderately), worrying about loved ones being affected by COVID-19 (very/extremely vs not at all/somewhat/moderately), feeling nervous and/or stressed (often/very often vs never/sometimes), and loneliness (≥5 vs <5 on the Three-Item Loneliness Scale^16^). The three questions on COVID-19-related feelings of worry, nervousness or stress were taken directly from the first version of the WHO Europe survey tool mentioned above. The Three-Item Loneliness Scale is a short version of the Revised UCLA Loneliness Scale (R-UCLA) that is part of several European ageing cohorts such as the Survey of Health, Ageing and Retirement in Europe (SHARE) or the English Longitudinal Study of Ageing (ELSA). It measures indirect loneliness based on the items ‘lack of companionship’, ‘left out’ and ‘isolated’, which are answered on a 3-point Likert scale (‘often’, ‘some of the time’, ‘hardly ever or never’). The minimum of the resulting score is 3 (‘not lonely’) and the maximum is 9 (‘very lonely’). Changes in social and physical activities were measured by asking participants about reductions in social interactions, reductions in light physical activity (yes/no) and reductions in vigorous physical activity (yes/no). A reduction in social interactions, hereon referred to as social isolation, was defined as a reduction in physical communication with family and friends without an increase in phone and/or video communication. Care-related items included refraining from seeking medical care (yes/no) and receiving reduced care at home. Reduced care at home was defined as a decrease in the use of formal home care services without an increase in received informal care.

### Preventive measures and sources of information

Participants were asked about their adherence to a list of nine recommendations—both general and those specific to the elderly—and the most common sources of information regarding the COVID-19 pandemic.

### Sociodemographics

Sociodemographic variables in the present study included age, sex, education and living arrangement. Age was dichotomised as youngest old (≤80 years old) and oldest old (>80 years old). Highest obtained education was dichotomised as low (elementary) and high (high school, university or above). Living arrangement was dichotomised into those who lived alone and those who did not.

### Statistical analysis

Characteristics of the study sample were reported as overall, as well as stratified by the four sociodemographic variables. Between-group differences were assessed via two-tailed t-tests and χ^2^ tests as appropriate. Binary logistic regression models were used to assess the associations between sociodemographic variables and the different collateral damage dimensions, as well as each item within these dimensions. All models were mutually adjusted for all sociodemographic variables. All statistical tests were performed using StataSE V.15 (StataCorp, College Station, Texas, USA). Significance level (alpha) was set at 0.05 for all analyses.

### Patient and public involvement

There was no direct public involvement either in the setting of the research questions or developing the study design.

## Results

The mean age of participants was 78.2 years, 64.3% were female, 34.3% had elementary educational attainment and 50.2% lived alone (table 1). Five per cent of participants (n=62) reported being tested for COVID-19, nine of which reported testing positive. Almost half of the sample (48.3%, n=595) sought medical care during the period March–June 2020, with 79 of them finding it more difficult to access it. Nine participants reported being hospitalised for suspected or confirmed COVID-19.

**Table 1:** Study sample characteristics (N=1231)

**Age, mean (SD)**	78.2 (8.3)
**Age, n (%)**	
≤80 years	642 (52.2)
>80 years	589 (47.8)
**Female, n (%)**	792 (64.3)
**Education, n (%)**	
High school, university or above	809 (65.7)
Elementary school	422 (34.3)
**Living alone, n (%)**	616 (50.2)
**COVID-19-related symptoms, n (%)**	
0	801 (65.1)
1	183 (14.9)
2+	247 (20.0)
**Tested for COVID-19, n (%)**	
Yes, positive	9 (0.7)
Yes, negative/unknown	53 (4.3)
**Sought medical care, n (%)**	595 (48.6)
Found it difficult to access healthcare services,* **n (%)**	79 (14.6)
**Hospitalised due to confirmed or suspected COVID-19, n (%)**	9 (0.7)

*Subsample of those who sought medical care (n=540).

**Figure 1:**
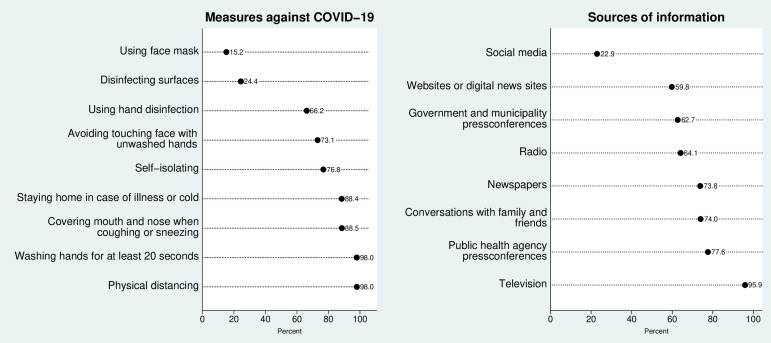
Adherence to preventive recommendations (left) and sources of information (right) related to COVID-19 during the first outbreak in Stockholm (March–June 2020).

The most commonly reported preventive behaviours were physical distancing of at least 2 m (98.0%) and washing hands for at least 20 s (98.0%), followed by covering mouth and nose when coughing or sneezing (88.5%) and staying home in case of illness or cold (88.4%) (figure 1). Three-quarters of the sample (76.8%) reported self-isolating. The least commonly reported measure was usage of face masks (15.2%). Most participants stayed up to date on the COVID-19 pandemic using television (95.9%); over three-quarters (77.6%) reported following the Public Health Agency’s press conferences. Digital sources, such as social media and online news websites, were the least reported sources (22.9% and 59.8%, respectively).

Half of the sample experienced certain level of psychological burden, with the most common items being loneliness (33.4%) and worrying about loved ones getting COVID-19 (24.9%) (table 2). More than half (55.3%) experienced a reduction in either social or physical activities, and 11.3% either refrained from seeking medical care or received less social care at home. In total, 77.8% of participants (n=956) experienced at least one of the three dimensions of collateral damage comprising psychological burden, reductions in social and physical activities, and decreased medical and social care use. Almost half (43.7%) reported experiencing one dimension of collateral damage, while the remainder (34.1%) experienced two or more. Univariate associations between each of the four sociodemographic variables and the different collateral damage dimensions are presented in online supplemental tables 1 and 2.

10.1136/bmjopen-2021-058422.supp1 Supplementary data

**Table 2:** Psychological burden and behavioural changes in the study sample (N=1231) during the first COVID-19 outbreak in Stockholm (March–June 2020)

Psychological burden, n (%)	
Worried about getting COVID-19	
Not at all, slightly, moderately	1032 (85.4)
Very, extremely	176 (14.6)
Worried about loved one getting COVID-19	
Not at all, slightly, moderately	907 (75.1)
Very, extremely	300 (24.9)
Felt nervous/stressed	
Never, sometimes	1112 (91.9)
Often, very often	98 (8.1)
Felt lonely*	
To a low extent (<5)	790 (66.6)
To a high extent (≥5)	396 (33.4)
Affected by at least one item	608 (49.8)
**Reductions in social and physical activities, n (%)**	
Social isolation†	195 (16.3)
Reduced light physical activity	352 (29.4)
Reduced vigorous physical activity	326 (27.3)
Affected by at least one item	676 (55.3)
**Reductions in care use, n (%)**	
Refrained from seeking medical care	126 (10.3)
Received less home care‡§	16 (8.9)
Affected by at least one item	139 (11.3)
**Sum of collateral damage dimensions, n (%)**	
0	273 (22.2)
1	537 (43.7)
2	371 (30.2)
3	48 (3.9)

*Based on the Three-Item Loneliness Scale (range: 3–9).

†Reduction in physical communication without an increase in phone and/or video communication.

‡Reduction in formal care without an increase in informal care.

§Subsample of those who received home care before the pandemic (n=180).

Women had higher odds of experiencing all items within the dimensions of psychological burden and social and physical activity reduction (table 3). Within the dimension of psychological burden, the oldest old had significantly lower odds of worrying about getting COVID-19. Those who lived alone had significantly lower odds of worrying about loved ones getting COVID-19, but higher odds of loneliness. Within the dimension of social and physical activity reductions, the oldest old had significantly higher odds of reducing light physical activity, while the oldest old and those who lived alone had lower odds of decreasing vigorous activity. Within the dimension of medical and social care use reduction, those with lower education had higher odds of receiving less care at home.

**Table 3:** Association (ORs and 95% CIs) between psychological burden and behavioural changes and sociodemographic factors (N=1231) during the first COVID-19 outbreak in Stockholm (March–June 2020)

	Oldest vs youngest old	P value	Female vs male	P value	Low vs high education*	P value	Living alone vs not living alone	P value
**Psychological burden**								
Worried about getting COVID-19	0.62 (0.44 to 0.87)	0.006	1.41 (0.99 to 2.03)	0.060	0.90 (0.64 to 1.28)	0.575	0.91 (0.65 to 1.29)	0.597
Worried about loved one getting COVID-19	0.84 (0.64 to 1.11)	0.215	1.54 (1.15 to 2.07)	0.004	0.99 (0.75 to 1.33)	0.632	0.63 (0.47 to 0.84)	0.001
Felt nervous/stressed	0.73 (0.47 to 1.13)	0.156	2.08 (1.23 to 3.52)	0.006	1.04 (0.66 to 1.62)	0.860	1.36 (0.87 to 2.16)	0.175
Felt lonely†	1.08 (0.84 to 1.40)	0.551	1.61 (1.22 to 2.12)	0.001	0.83 (0.64 to 1.09)	0.185	1.50 (1.15 to 1.95)	0.003
Affected by at least one item	0.94 (0.74 to 1.19)	0.597	1.90 (1.48 to 2.45)	<0.001	0.97 (0.75 to 1.24)	0.777	1.13 (0.89 to 1.45)	0.320
**Reductions in social and physical activity**								
Social isolation‡	1.19 (0.86 to 1.63)	0.295	0.64 (0.46 to 0.89)	0.008	1,31 (0.95 to 1.82)	0.104	1.13 (0.81 to 1.58)	0.476
Reduced light physical activity	1.82 (1.40 to 2.37)	<0.001	1.58 (1.19 to 2.11)	0.002	0.94 (0.72 to 1.24)	0.680	1.06 (0.80 to 1.39)	0.692
Reduced vigorous physical activity	0.55 (0.42 to 0.72)	<0.001	1.32 (0.99 to 1.76)	0.056	0.79 (0.60 to 1.06)	0.116	0.76 (0.57 to 1.00)	0.05
Affected by at least one item	1.17 (0.93 to 1.49)	0.181	1.30 (1.08 to 1.78)	0.009	1.05 (0.82 to 1.34)	0.691	0.89 (0.70 to 1.14)	0.373
**Reductions in care use**								
Refrained from seeking medical care	0.98 (0.67 to 1.44)	0.928	1.05 (0.70 to 1.58)	0.801	0.94 (0.63 to 1.40)	0.761	1.1 (0.74 to 1.64)	0.637
Received less home care§¶	0.14 (0.03 to 0.62)	0.010	3.58 (0.63 to 20.4)	0.151	3.98 (1.15 to 13.7)	0.029	1.31 (0.30 to 5.63)	0.718
Affected by at least one item	1.06 (0.73 to 1.53)	0.757	1.10 (0.74 to 1.63)	0.643	1.11 (0.76 to 1.62)	0.595	1.15 (0.79 to 1.69)	0.468

Models mutually adjusted by all sociodemographic factors.

*Low=elementary, high=high school, university or above.

†Based on the Three-Item Loneliness Scale (range: 3–9).

‡Defined as reduction in physical communication without an increase in phone and/or video communication.

§Defined as reduction in formal care without an increase in informal care.

¶Subsample of those who received home care before the pandemic (n=180).

**Figure 2:**
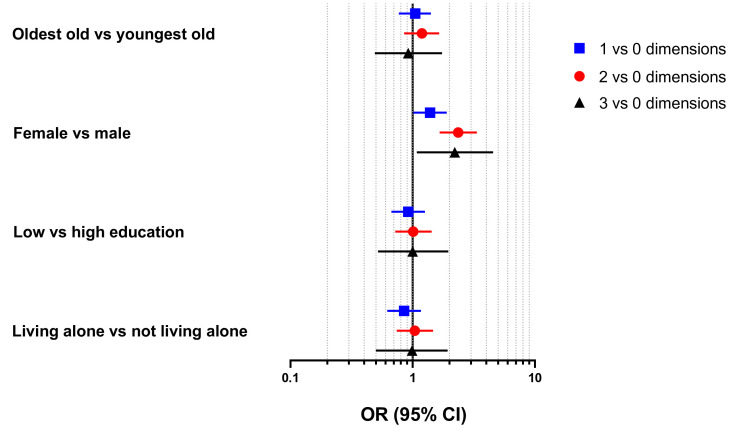
Association (ORs and 95% CIs) between number of experienced dimensions of collateral damage and sociodemographic factors (N=1231) during the first COVID-19 outbreak in Stockholm (March–June 2020). Models mutually adjusted by all sociodemographic factors.

Women were more likely to experience one (OR: 1.38, 95% CI: 1.01 to 1.90), two (OR: 2.36, 95% CI: 1.66 to 3.35) and all three (OR: 2.21, 95% CI: 1.08 to 4.55) collateral damage dimensions compared with men (figure 2). No statistically significant differences were detected for age, education and living arrangement in terms of the number of experienced dimensions of collateral damage, after adjusting for the rest of sociodemographic factors.

## Discussion

In this study examining the collateral damage of COVID-19 restrictions in terms of psychological burden, reductions in social and physical activities, and decreased medical and social care use in a Swedish urban older population, we found that over three-quarters of the sample were affected by at least one dimension, with women being at a considerably higher risk. We also showed that the vast majority of the study population adhered to the COVID-19 preventive measures during the first half of 2020.

### Interpretation of results

Adherence to national recommendations is an important factor in mitigating the negative effects of the COVID-19 pandemic. We found that participants in our study were well informed about the pandemic and adopted low-risk behaviours during the first wave of the COVID-19 outbreak. The majority of participants followed the Public Health Agency press conferences, likely reflecting the high social and institutional trust in Sweden,^14^ and adhered to the agency’s strongly recommended preventive measures.

We observe a substantial impact of the pandemic on the mental health of the elderly, with half of the sample reporting psychological burden. A fair share of the sample reported worrying about themselves and their loved ones being affected by COVID-19. Interestingly, the latter seems to be of more concern, a finding that has been replicated in another Swedish survey.^14^ Loneliness and feelings of stress were also prevalent in our sample. This is in line with a large body of research showing considerable effects of the pandemic on mental health outcomes.^17 18^ The burden seems to be unevenly borne by women and, to a lesser extent, by those living alone. Indeed, previous research has shown that women are at a higher risk of poor mental health^18^ and worrying about family^19^ during the pandemic.

High adherence to self-isolation recommendations, combined with a decrease in physical contact with loved ones, puts older adults at risk of social isolation. Social isolation presents a major modern-day challenge and has been associated with several negative health outcomes, such as depression,^20^ frailty,^21^ cognitive decline^22^ and low quality of life.^23 24^ While we did observe a reduction in frequency of physical meetings with family, friends and neighbours, this was largely met with an increase in phone and video communication with them. This is very important in the context of preventing the negative effects of loneliness and social isolation, as alternate forms of communication may buffer such effects.^25 26^

Concern has been raised about reduction in physical activity as a major collateral consequence of the pandemic restrictions, since low physical activity is linked to negative cardiovascular and metabolic outcomes,^9^ poor mental health,^10^ frailty^27^ and insomnia,^28^ among others. In spite of the Public Health Agency’s recommendations for the elderly to remain physically active and spend time outdoors in a safe manner, we still found that up to one-third of the sample had decreased their frequency of both light and vigorous physical activity. Furthermore, it is important to highlight that the reduction in light physical activity was more prominent among the oldest old, who, in all likelihood, were also doing less incidental physical activity, such as climbing the stairs or visiting the supermarket, due to self-isolation. They might encounter difficulties in returning to their former activity levels should the pandemic persist for a long time, which requires close monitoring from medical and social services.

We found that subjects in our sample limited their contact with the healthcare system during the first wave of the pandemic, but when seeking for help, received it in a timely and satisfactory way. This is an important finding in a context where hospital overcrowding has emerged as an important challenge in many countries. Around 10% of our sample refrained from seeking medical care altogether, which may explain, among others, the reduction in cancer diagnosis by Swedish healthcare services compared with previous years.^29^ Still, the proportion of those refraining was significantly lower than in the USA, where one-third of the population aged 65+ years reported delaying or avoiding medical care during the first wave of the pandemic.^30^ Subjects also decreased their use of formal care but seem to have compensated for it by an increase in received informal care. This could become a concern should the pandemic continue, since it is widely acknowledged that informal caregiving places significant economic, physical and mental burden on caregivers, who are often older adults with healthcare needs of their own.^31^

### Strengths and limitations

To the best of our knowledge, this study is the first to examine the consequences of the Swedish COVID-19 strategy in a random sample of urban older adults. Further strengths include the use of an ad hoc questionnaire developed by a multidisciplinary team of experts, and the study sample coming from a well-characterised population-based study. Being based on data from Sweden, the study also provides a unique opportunity for comparison with other settings, as the Swedish strategy against COVID-19 differed from most countries. Certain limitations also need to be highlighted. We did not have recent pre-pandemic measures of physical and mental health. Thus, despite participants being asked to answer the questions for the period since March 2020, the cross-sectional study design does not allow us to assess the temporal relationship between the onset of the pandemic and the studied outcomes. The findings from this cohort of older adults living in an affluent neighbourhood of Stockholm may not be generalisable to the entire Swedish population. However, these findings could be viewed as a best-case scenario, and the identified collateral damage may be expected to be of a higher magnitude in less urban and affluent parts of the country.

## Conclusion

The results from this study indicate that, in addition to an excess mortality, COVID-19 and its related restrictions during the first half of 2020 have also resulted in changes that negatively affect the health and lives of the elderly living in central Stockholm. Furthermore, we found age-, sex-, living arrangement- and, to a much lesser extent, education-related differences in the susceptibility to these consequences, with women being at a particularly increased risk. When introducing restrictions, we emphasise the need for a predefined, evidence-based strategy to provide support, both during the pandemic and once the outbreak is overcome, to those who are most susceptible to these consequences.

## Supplementary Material

Reviewer comments

Author's manuscript

## Data Availability

Data are available upon reasonable request. Data are from the SNAC-K Project, a population-based study on ageing and dementia (http://www.snac-k.se/). Access to these original data is available to the research community upon approval by the SNAC-K data management and maintenance committee. Applications for accessing these data can be submitted to Maria Wahlberg (Maria.Wahlberg@ki.se) at the Aging Research Center, Karolinska Institutet.
