# Supplementary material for: COVID-19 collateral damage—psychological burden and behavioural changes among older adults during the first outbreak in Stockholm, Sweden: a cross-sectional study
Source: BMJ Open. 2022 Jan 7;12(1):e058422. doi: 10.1136/bmjopen-2021-058422 (PMC8743841; doi:10.1136/bmjopen-2021-058422)
Supplement: Supplementary data [file bmjopen-2021-058422supp001.pdf]

Supplementary Table 1. Psychological burden and behavioral changes in the study sample (N=1231) by age and sex during the first COVID-19 outbreak in Stockholm (March-June 2020).

	**Age**		*p-value*	**Sex**		*p-value*
	Under 80	Over 80		Male	Female	
	n=809	n=422		n=439	n=792	
**Psychological burden**						
**Worried about getting COVID-19**			0.003			0.13
Not at all, slightly, moderately	522 (82.6%)	510 (88.5%)		378 (87.5%)	654 (84.3%)	
Very, extremely	110 (17.4%)	66 (11.5%)		54 (12.5%)	122 (15.7%)	
**Worried about loved one getting COVID-19**			0.054			0.063
Not at all, slightly, moderately	462 (72.9%)	445 (77.7%)		338 (78.2%)	569 (73.4%)	
Very, extremely	172 (27.1%)	128 (22.3%)		94 (21.8%)	206 (26.6%)	
**Felt nervous/stressed**			0.45			0.002
Never, sometimes	580 (91.3%)	532 (92.5%)		410 (95.1%)	702 (90.1%)	
Often or very often	55 (8.7%)	43 (7.5%)		21 (4.9%)	77 (9.9%)	
**Felt lonely**[a]			0.19			<0.001
To a low extent (<5)	429 (68.3%)	361 (64.7%)		319 (74.7%)	471 (62.1%)	
To a high extent (≥5)	199 (31.7%)	197 (35.3%)		108 (25.3%)	288 (37.9%)	
**Affected by at least one item**	318 (49.7%)	290 (49.8%)	0.96	171 (39.1%)	437 (55.7%)	<0.001
**Reductions in social and physical activities**						
**Social isolation**[b]	94 (14.9%)	101 (17.7%)	0.19	97 (22.8%)	255 (33.0%)	<0.001
**Reduced light physical activity**	145 (23.2%)	207 (36.2%)	<0.001	110 (26.0%)	216 (28.1%)	0.45
**Reduced vigorous physical activity**	209 (33.6%)	117 (20.5%)	<0.001	220 (50.5%)	456 (58.0%)	0.011
**Affected by at least one item**	341 (53.3%)	335 (57.6%)	0.13	83 (19.4%)	112 (14.5%)	0.025
**Reductions in care use**						
**Refrained from seeking medical care**	66 (10.3%)	60 (10.3%)	0.99	43 (9.9%)	83 (10.5%)	0.71
**Received less home care**[c,d]	4 (22.2%)	12 (7.4%)	0.036	2 (4.1%)	14 (10.7%)	0.17
**Affected by at least one item**	69 (10.8%)	70 (11.9%)	0.52	45 (10.3%)	94 (11.9%)	0.40
**Sum of collateral damage dimensions**						
**0**	147 (22.9%)	126 (21.4%)	0.40	123 (28.1%)	150 (19.0%)	<0.001
**1**	287 (44.8%)	250 (42.5%)		207 (47.3%)	330 (41.7%)	
**2**	180 (28.1%)	191 (32.5%)		95 (21.7%)	276 (34.9%)	
**3**	27 (4.2%)	21 (3.6%)		13 (3.0%)	35 (4.4%)	

[a] Based on the Three-Item Loneliness Scale (range: 3-9)
[b] Reduction in physical communication without an increase in phone and/or video communication
[c] Reduction in formal care without an increase in informal care
[d] Subsample of those who received home care before the pandemic (n=180)

Supplementary Table 2. Psychological burden and behavioral changes in the study sample (N=1231) by education and living arrangement during the first COVID-19 outbreak in Stockholm (March-June 2020).

	Education[a]		*p-value*	Living arrangement		*p-value*
	Low	High		Living alone	Not living alone	
	n=422	n=809		n=612	n=616	
**Psychological burden**						
**Worried about getting COVID-19**			0.35			0.46
Not at all, slightly, moderately	360 (86.7%)	672 (84.7%)		508 (84.7%)	523 (86.2%)	
Very, extremely	55 (13.3%)	121 (15.3%)		92 (15.3%)	84 (13.8%)	
**Worried about loved one getting COVID-19**			0.66			0.004
Not at all, slightly, moderately	312 (75.9%)	595 (74.7%)		433 (71.6%)	473 (78.7%)	
Very, extremely	99 (24.1%)	201 (25.3%)		172 (28.4%)	128 (21.3%)	
**Felt nervous/stressed**			0.62			0.042
Never, sometimes	381 (91.4%)	731 (92.2%)		566 (93.6%)	545 (90.4%)	
Often or very often	36 (8.6%)	62 (7.8%)		39 (6.4%)	58 (9.6%)	
**Felt lonely[b]**			0.81			<0.001
To a low extent (<5)	269 (67.1%)	521 (66.4%)		431 (72.4%)	358 (60.8%)	
To a high extent (≥5)	132 (32.9%)	264 (33.6%)		164 (27.6%)	231 (39.2%)	
**Affected by at least one item**	213 (50.7%)	395 (49.3%)	0.63	280 (46.1%)	326 (53.3%)	0.012
**Reductions in social and physical activities**						
**Social isolation[c]**	76 (18.6%)	119 (15.0%)	0.11	95 (15.8%)	100 (16.7%)	0.67
**Reduced light physical activity**	129 (31.2%)	223 (28.4%)	0.31	156 (26.2%)	196 (32.7%)	0.013
**Reduced vigorous physical activity**	94 (22.9%)	232 (29.7%)	0.012	183 (31.0%)	142 (23.7%)	0.005
**Affected by at least one item**	239 (57.0%)	437 (54.4%)	0.38	334 (54.9%)	341 (55.7%)	0.78
**Reductions in care use**						
**Refrained from seeking medical care**	42 (10.1%)	84 (10.4%)	0.85	60 (9.9%)	66 (10.8%)	0.61
**Received less home care[d,e]**	12 (14%)	4 (4%)	0.025	3 (6.7%)	13 (9.6%)	0.55
**Affected by at least one item**	52 (12.4%)	87 (10.8%)	0.41	63 (10.3%)	76 (12.4%)	0.27
**Sum of collateral damage dimensions**						
**0**	92 (21.8%)	181 (22.4%)	0.38	141 (23.1%)	132 (21.4%)	0.046
**1**	173 (41.0%)	364 (45.1%)		285 (46.6%)	251 (40.7%)	
**2**	140 (33.2%)	231 (28.6%)		163 (26.7%)	207 (33.6%)	
**3**	17 (4.0%)	31 (3.8%)		22 (3.6%)	26 (4.2%)	

[a] Low = elementary, high = high school, university or above
[b] Based on the Three-Item Loneliness Scale (range: 3-9)
[c] Reduction in physical communication without an increase in phone and/or video communication
[d] Reduction in formal care without an increase in informal care
[e] Subsample of those who received home care before the pandemic (n=180)
