# Supplementary material for: COVID-19 collateral damage—psychological burden and behavioural changes among older adults during the first outbreak in Stockholm, Sweden: a cross-sectional study
Source: BMJ Open. 2022 Jan 7;12(1):e058422. doi: 10.1136/bmjopen-2021-058422 (PMC8743841; doi:10.1136/bmjopen-2021-058422)
Supplement: Author's manuscript [file bmjopen-2021-058422.draft_revisions.pdf]

BMJ Open is committed to open peer review. As part of this commitment we make the peer review history of every article we publish publicly available.

When an article is published we post the peer reviewers' comments and the authors' responses online. We also post the versions of the paper that were used during peer review. These are the versions that the peer review comments apply to.

The versions of the paper that follow are the versions that were submitted during the peer review process. They are not the versions of record or the final published versions. They should not be cited or distributed as the published version of this manuscript.

BMJ Open is an open access journal and the full, final, typeset and author-corrected version of record of the manuscript is available on our site with no access controls, subscription charges or pay-per-view fees (http://bmjopen.bmj.com).

If you have any questions on BMJ Open's open peer review process please email info.bmjopen@bmj.com

BMJ Open

COVID-19 collateral damage: psychological burden and behavioral changes among older adults during the first outbreak in Stockholm, Sweden

Journal:	*BMJ Open*
Manuscript ID	bmjopen-2021-058422
Article Type:	Original research
Date Submitted by the Author:	15-Oct-2021

Complete List of Authors:	Beridze, Giorgi; Karolinska Institute, Aging Research Center, Department of Neurobiology, Care Sciences and Society<br>Triolo, Federico; Karolinska Institute, Aging Research Center, Department of Neurobiology, Care Sciences and Society<br>Grande, Giulia; Karolinska Institute, Aging Research Center, Department of Neurobiology, Care Sciences and Society<br>Fratiglioni, Laura; Karolinska Institute, Aging Research Center, Department of Neurobiology, Care Sciences and Society; Stockholm Gerontology Research Center<br>Calderón-Larrañaga, Amaia; Karolinska Institute, Aging Research Center, Department of Neurobiology, Care Sciences and Society

Keywords:	COVID-19, EPIDEMIOLOGY, GERIATRIC MEDICINE

SCHOLARONE™
Manuscripts

I, the Submitting Author has the right to grant and does grant on behalf of all authors of the Work (as defined in the below author licence), an exclusive licence and/or a non-exclusive licence for contributions from authors who are: i) UK Crown employees; ii) where BMJ has agreed a CC-BY licence shall apply, and/or iii) in accordance with the terms applicable for US Federal Government officers or employees acting as part of their official duties; on a worldwide, perpetual, irrevocable, royalty-free basis to BMJ Publishing Group Ltd ("BMJ") its licensees and where the relevant Journal is co-owned by BMJ to the co-owners of the Journal, to publish the Work in this journal and any other BMJ products and to exploit all rights, as set out in our licence.

The Submitting Author accepts and understands that any supply made under these terms is made by BMJ to the Submitting Author unless you are acting as an employee on behalf of your employer or a postgraduate student of an affiliated institution which is paying any applicable article publishing charge ("APC") for Open Access articles. Where the Submitting Author wishes to make the Work available on an Open Access basis (and intends to pay the relevant APC), the terms of reuse of such Open Access shall be governed by a Creative Commons licence – details of these licences and which Creative Commons licence will apply to this Work are set out in our licence referred to above.

Other than as permitted in any relevant BMJ Author's Self Archiving Policies, I confirm this Work has not been accepted for publication elsewhere, is not being considered for publication elsewhere and does not duplicate material already published. I confirm all authors consent to publication of this Work and authorise the granting of this licence.

For peer review only

TITLE

COVID-19 collateral damage: psychological burden and behavioral changes among older adults during the first outbreak in Stockholm, Sweden

AUTHORS

Giorgi Beridze[1], Federico Triolo[1], Giulia Grande[1], Laura Fratiglioni[1,2], Amaia Calderón-Larrañaga[1]

AFFILIATIONS

1. Aging Research Center, Department of Neurobiology, Care Sciences and Society, Karolinska Institutet-Stockholm University, 17165 Solna, Sweden
2. Stockholm Gerontology Research Center, Stiftelsen Stockholms läns Äldrecentrum, 113 46 Stockholm, Sweden

CORRESPONDING AUTHOR

Giorgi Beridze, MD, MMSc

Tomtebodavägen 18a, Solna, Sweden 17165

giorgi.beridze@ki.se

KEYWORDS

COVID-19; collateral damage; restrictions; older adults

WORD COUNT: 2749

For peer review only

ABSTRACT

Introduction: During the first wave of the COVID-19 pandemic, Swedish public health authorities provided recommendations for 70+ year old people. They were strongly encouraged to self-isolate but remain physically active in a safe manner. This study aimed to explore the indirect, negative effects of COVID-19 restrictions (collateral damage) on the lives and health of older adults living in central Stockholm, and to characterize the sociodemographic profile of those with the highest susceptibility to this damage.

Methods: An *ad-hoc* phone questionnaire was administered by trained staff between May and June 2020 to a random sample of older adults 68+ years old (n=1231), who had attended the regular follow-up assessment of the Swedish National study on Aging and Care in Kungsholmen (SNAC-K) during 2016-2019. We explored three dimensions of collateral damage: psychological burden (feelings of worry, stress and loneliness), reductions in social and physical activities, and reductions in medical and social care use since the beginning of the pandemic. Logistic regression models were used to test the association between age, sex, education and living arrangement, and the risk of collateral damage.

Results: Vast majority of participants adhered to the recommendations, with over three quarters practicing self-isolation (n=928). Half of the sample reported psychological burden, 55.3% reported reductions in social or physical activity, and 11.3% reported decreased medical or social care use. 77.8% were affected by at least one of the three collateral damage dimensions. Female sex was the strongest sociodemographic predictor of both individual and co-occurring dimensions of collateral damage.

Conclusion: COVID-19 and its restrictions during the first half of 2020 had a negative effect on the health and lives of a majority of elderly living in central Stockholm. Women were at a

For peer review only

higher risk of these negative consequences. We emphasize the need for predefined, evidence-based interventions to address these negative consequences.

STRENGTHS AND LIMITATIONS OF THIS STUDY

- This study uses a large sample of older adults from a well-characterized population-based study (SNAC-K)
- Several dimensions of the indirect, negative effects (collateral damage) of the COVID-19 pandemic are explored in this study
- As Sweden's response to COVID-19 differed from most countries, this study provides a unique opportunity of comparison with other settings
- The cross-sectional design of this study does not allow to establish temporality between the onset of the pandemic and studied outcomes
- The results of this study may not be generalizable to the entire elderly population in Sweden as participants are from an urban, affluent area in Stockholm

For peer review only

For peer review only - http://bmjopen.bmj.com/site/about/guidelines.xhtml

INTRODUCTION

The outbreak caused by the novel coronavirus, SARS-CoV2, was declared a global pandemic by the World Health Organization (WHO) on March 11th 2020, coinciding with the date of the first confirmed death in Sweden. Early on, it was identified that older adults were at a significantly higher risk of mortality from COVID-19. Indeed, as of January 18th 2021, 91% of deaths attributed to COVID-19 in Sweden have happened in those aged 70 and above [1]. Later on, additional prognostic factors were identified, including male sex, socioeconomic disadvantage, the presence of comorbidities and frailty [2–5].

In response to the pandemic, most countries have implemented strict measures to help curb the spread of the virus and reduce mortality. Sweden's response to COVID-19 differed from most countries by not implementing strict lockdowns and restrictions, but instead relying on high voluntary adherence to the recommendations proposed by the Public Health Agency. The general recommendations included keeping good hand hygiene, practicing social distancing and avoiding contact if having any symptoms [6]. On top of these, the specific recommendations for the elderly were to stay at home, avoid social gatherings and public transportation, but to remain physically active outdoors in a safe manner [6].

The importance of looking beyond mortality and morbidity when assessing national response strategies for COVID-19 has been raised [7]. Stay-at-home orders and lack of contact with loved ones put elderly at risk of loneliness and social isolation, which, in turn, are known to have negative effects, particularly in old age [8]. Reduced physical activity and sedentarism have detrimental effects on physical and mental health [9,10]. Additionally, due to the overburdening of healthcare services and reduced access to medical, social and informal care, new conditions may not be timely diagnosed, and existing health conditions may be exacerbated. These consequences can be considered indirect, negative effects (i.e. collateral damage) of COVID-19 restrictions.

For peer review only - http://bmjopen.bmj.com/site/about/guidelines.xhtml

While several studies have examined the distribution of COVID-19 mortality among Swedish older adults by sex, socioeconomic and household factors [11–13], little is known on the collateral damage of these restrictions. To the best of our knowledge, only one study has examined the mental health consequences of the Swedish strategy on the elderly [14], and no study has looked into other dimensions such as psychological wellbeing and/or behavioral changes. Thus, the aims of this study were to explore different dimensions of the collateral damage linked to COVID-19 during the first epidemic outbreak in an older population of central Stockholm, as well as to characterize the sociodemographic profile of those with the highest susceptibility to this damage.

METHODS

Study population

Study population consisted of 1231 older adults aged between 68 and 103, participating in the Swedish National study on Aging and Care in Kungsholmen (SNAC-K). SNAC-K is a longitudinal study including a random sample of older adults aged 60 years and above living in the Kungsholmen area of Stockholm, Sweden. All SNAC-K participants who had participated in the regular follow-up assessment during 2016-2019 were invited to participate in a telephone interview aimed at monitoring preventive behaviors and the direct and indirect health consequences of the COVID-19 pandemic. Telephone interviews were conducted between May and June 2020 (95%) by trained SNAC-K staff, following a multi-choice questionnaire that was *ad hoc* developed by the SNAC-K data collection team as well as experts in geriatric medicine, neurology and public health. The questionnaire comprised a selection of items from the original SNAC-K assessments and from the WHO Europe survey tool to monitor the public's risk perceptions, behaviors, trust and knowledge concerning the pandemic outbreak response [15]. The interview was preceded by a brief explanation whereby participants were told that all

For peer review only - http://bmjopen.bmj.com/site/about/guidelines.xhtml

questions referred specifically to the pandemic context. Exclusion criteria included living in care and nursing homes, known diagnosis of dementia and very impaired hearing. The response rate was 91.9%. Subjects who refused to participate or could not be contacted had similar age and educational attainment to those who participated, but were more likely to be male (45.4% vs 35.7%, p=0.044).

Collateral damage

In this study we examined three dimensions of collateral damage: psychological burden and two aspects related to behavioral changes, i.e. reductions in social and physical activities, and in medical and social care use. All questions explicitly asked participants about changes since the beginning of the pandemic. Psychological burden was assessed with variables related to worrying about being affected by COVID-19 (very/extremely vs. not at all/somewhat/moderately), worrying about loved ones being affected by COVID-19 (very/extremely vs. not at all/somewhat/moderately), feeling nervous and/or stressed (often/very often vs. never/sometimes), and loneliness (≥5 vs. <5 on the Three-Item Loneliness Scale [16]). The three questions on COVID19-related feelings of worry, nervousness or stress were taken directly from the first version of the WHO Europe survey tool mentioned above. The Three-Item Loneliness Scale is a short version of the R-UCLA Loneliness Scale that is part of several European aging cohorts such as the Survey of Health, Ageing and Retirement in Europe (SHARE) or the English Longitudinal Study of Ageing (ELSA). It measures indirect loneliness based on the items “companionship”, “left out”, and “isolated”, which are answered on a three point Likert scale (“often”, “some of the time”, “hardly ever or never”). The minimum of the resulting score is 3 (“not lonely”) and the maximum is 9 (“very lonely”). Changes in social and physical activities were measured by asking participants about reductions in social interactions, reductions in light physical activity (yes/no) and reductions in vigorous physical activity (yes/no). A reduction in social interactions, hereon referred to as social isolation, was

For peer review only - http://bmjopen.bmj.com/site/about/guidelines.xhtml

defined as a reduction in physical communication with family and friends without an increase in phone and/or video communication. Care-related items included refraining from seeking medical care (yes/no) and receiving reduced care at home. Reduced care at home was defined as a decrease in the use of formal home-care services without an increase in received informal care.

Preventive measures and sources of information

Participants were asked about their adherence to a list of 9 recommendations −both general and those specific to elderly−, and the most common sources of information regarding the COVID-19 pandemic.

Sociodemographics

Sociodemographic variables in the present study included age, sex, education and living arrangement. Age was dichotomized as youngest old (≤80 years old) and oldest old (>80 years old). Highest obtained education was dichotomized as low (elementary) and high (high school, university, or above). Living arrangement was dichotomized into those who lived alone and those who did not.

Statistical analysis

Characteristics of the study sample were reported as overall, as well as stratified by the four sociodemographic variables. Between-group differences were assessed via two-tailed t-tests and chi-square tests as appropriate. Binary logistic regression models were used to assess the associations between sociodemographic variables and the different collateral damage dimensions, as well as each item within these dimensions. All models were mutually adjusted for all sociodemographic variables. All statistical tests were performed in StataSE 15 (StataCorp LLC, College Station, TX, USA). Significance level (alpha) was set at 0.05 for all analyses.

For peer review only - http://bmjopen.bmj.com/site/about/guidelines.xhtml

Ethical considerations

Informed consent was obtained from all participants. The study was approved by the Regional Ethics Review Board in Stockholm (dnr: 2020-02497).

Patient and public involvement

There was no direct public involvement either in the setting of the research questions or developing the study design.

RESULTS

The mean age of participants was 78.2 years, 64.3% were female, 34.3% had elementary educational attainment and 50.2% lived alone (**Table 1**). Five percent of participants (n=62) reported being tested for COVID-19, 9 of which reported testing positive. Almost half of the sample (48.3%, n=595) sought medical care during the period March-June 2020, with 79 of them finding it more difficult to access it. Nine participants reported being hospitalized for suspected or confirmed COVID-19.

The most commonly reported preventive behaviors were physical distancing of at least two meters (98.0%) and washing hands for at least 20 seconds (98.0%), followed by covering mouth and nose when coughing or sneezing (88.5%) and staying home in case of illness or cold (88.4%) (**Figure 1**). Three quarters of the sample (76.8%) reported self-isolating. The least commonly reported measure was usage of face masks (15.2%). Most participants stayed up to date on the COVID-19 pandemic using television (95.9%); over three quarters (77.6%) reported following the Public Health Agency's press-conferences. Digital sources, such as social media and online news websites were the least reported sources (22.9% and 59.8% respectively).

For peer review only - http://bmjopen.bmj.com/site/about/guidelines.xhtml

Half of the sample experienced certain level of psychological burden, with the most common items being loneliness (33.4%) and worrying about loved ones getting COVID-19 (24.9%) (**Table 2**). More than half (55.3%) experienced a reduction in either social or physical activities, and 11.3% either refrained from seeking medical care or received less social care at home. In total, 77.8% of participants (n=956) experienced at least one of the three dimensions of collateral damage comprising psychological burden, reductions in social and physical activities, and decreased medical and social care use. Almost half (43.7%) reported experiencing one dimension of collateral damage, while the remainder (34.1%) experienced two or more. Univariate associations between each of the four sociodemographic variables and the different collateral damage dimensions are presented in **Supplementary Tables 1** and **2.**

Women had higher odds of experiencing all items within the dimensions of psychological burden and social and physical activity reduction (**Table 3**). Within the dimension of psychological burden, the oldest old had significantly lower odds of worrying about getting COVID-19. Those who lived alone had significantly lower odds of worrying about loved ones getting COVID-19, but higher odds of loneliness. Within the dimension of social and physical activity reductions, the oldest old had significantly higher odds of reducing light physical activity, while the oldest old and those who lived alone had lower odds of decreasing vigorous activity. Within the dimension of medical and social care use reduction, those with lower education had higher odds of receiving less care at home.

Women were more likely to experience one (OR: 1.38, 95% CI: 1.01;1.90), two (OR: 2.36, 95% CI: 1.66;3.35) and all three (OR: 2.21, 95% CI: 1.08;4.55) collateral damage dimensions compared to men (**Figure 2**). No statistically significant differences were detected for age, education and living arrangement in terms of the number of experienced dimensions of collateral damage, after adjusting for the rest of sociodemographic factors.

For peer review only - http://bmjopen.bmj.com/site/about/guidelines.xhtml

DISCUSSION

In this study examining the collateral damage of COVID-19 restrictions in terms of psychological burden, reductions in social and physical activities, and decreased medical and social care use in a Swedish urban older population, we found that over three quarters of the sample was affected by at least one dimension, with women being at a considerably higher risk. We also showed that the vast majority of the study population adhered to the COVID-19 preventive measures during the first half of 2020.

Interpretation of results

Adherence to national recommendations is an important factor in mitigating the negative effects of the COVID-19 pandemic. We found that participants in our study were well-informed about the pandemic and adopted low-risk behaviors during the first wave of COVID-19 outbreak. The majority of participants followed the Public Health Agency press-conferences, likely reflecting the high social and institutional trust in Sweden [14], and adhered to the Agency's strongly recommended preventive measures.

We observe a substantial impact of the pandemic on the mental health of the elderly, with half of the sample reporting psychological burden. A fair share of the sample reported worrying about themselves and their loved ones being affected by COVID-19. Interestingly, the latter seems to be of more concern, a finding that has been replicated in another Swedish survey [14]. Loneliness and feelings of stress were also prevalent in our sample. This is in line with a large body of research showing considerable effects of the pandemic on mental health outcomes [17,18]. The burden seems to be unevenly borne by women and, to a lesser extent, by those living alone. Indeed, previous research has shown that women are at a higher risk of poor mental health [18] and worrying about family [19] during the pandemic.

For peer review only - http://bmjopen.bmj.com/site/about/guidelines.xhtml

For peer review only

High adherence to self-isolation recommendations, combined with a decrease in physical contact with loved ones, puts older adults at risk of social isolation. Social isolation presents a major modern-day challenge and has been associated with several negative health outcomes, such as depression [20], frailty [21], cognitive decline [22] and low quality of life [23,24]. While we did observe a reduction in frequency of physical meetings with family, friends and neighbors, this was largely met with an increase in phone and video communication with them. This is very important in the context of preventing the negative effects of loneliness and social isolation, as alternate forms of communication may buffer such effects [25,26].

Concern has been raised about reduction in physical activity as a major collateral consequence of the pandemic restrictions, as low physical activity is linked to negative cardiovascular and metabolic outcomes [9], poor mental health [10], frailty [27], and insomnia [28], among others. In spite of the Public Health Agency's recommendations for the elderly to remain physically active and spend time outdoors in a safe manner, we still found that up to a third of the sample had decreased their frequency of both light and vigorous physical activity. Furthermore, it is important to highlight that the reduction in light physical activity was more prominent among the oldest old, who, in all likelihood, were also doing less incidental physical activity, such as climbing the stairs or visiting the supermarket, due to self-isolation. They might encounter difficulties in returning to their former activity levels should the pandemic persist for a long time, which requires close monitoring from medical and social services.

We found that subjects in our sample limited their contact with the healthcare system during the first wave of the pandemic, but when seeking for help, received it in a timely and satisfactory way. This is an important finding in a context where hospital overcrowding has emerged as an important challenge in many countries. Around 10% of our sample refrained from seeking medical care altogether, which may explain, among others, the reduction in cancer diagnosis by Swedish healthcare services compared to previous years [29]. Still, the proportion of those

For peer review only - http://bmjopen.bmj.com/site/about/guidelines.xhtml

refraining was significantly lower than in the US, where a third of the population aged 65+ reported delaying or avoiding medical care during the first wave of the pandemic [30]. Subjects also decreased their use of formal care but seem to have compensated for it by an increase in received informal care. This could become a concern should the pandemic persist, since it is widely acknowledged that informal caregiving places significant economic, physical and mental burden on caregivers, who are often themselves older adults with health needs [31].

Strengths and limitations

To the best of our knowledge, this study is the first to examine the consequences of the Swedish COVID-19 strategy in a random sample of urban older adults. Further strengths include the use of an *ad hoc* questionnaire developed by a multidisciplinary team of experts, and the study sample coming from a well-characterized population-based study. Being based on data from Sweden, the study also provides a unique opportunity for comparison with other settings, as the Swedish strategy against COVID-19 differed from most countries. Certain limitations also need to be highlighted. We did not have recent pre-pandemic measures of physical and mental health. Thus, despite participants being asked to answer the questions for the period since March, the cross-sectional design does not allow us to assess temporal relationship between the onset of the pandemic and the studied outcomes. The findings from this cohort of older adults living in an affluent neighborhood of Stockholm may not be generalizable to the entire Swedish population. However, these findings could be viewed as a best-case scenario, and the identified collateral damage would be expected to be of higher magnitude in less urban and affluent parts of the country.

Conclusion

The results from this study indicate that, in addition to morbidity and mortality, COVID-19 and its related restrictions during the first half of 2020 have also resulted in changes that negatively

For peer review only - http://bmjopen.bmj.com/site/about/guidelines.xhtml
For peer review only

affect the health and lives of the elderly living in Central Stockholm. Furthermore, we found age-, sex-, living arrangement- and, to a much lesser extent, education-related differences in the susceptibility to these consequences, with women being at a particularly increased risk. When introducing restrictions, we emphasize the need of a predefined, evidence-based strategy to provide support, both during the pandemic and once the outbreak is overcome, to those who are most susceptible to these consequences.

For peer review only

Contributors: GB, LF, and AC-L developed the study concept and design. GB performed the data analysis and drafted the manuscript. All authors interpreted the data, provided critical revisions and approved the final version of the manuscript for submission.

Funding: This work was supported by the funders of the Swedish National study on Aging and Care (SNAC): the Ministry of Health and Social Affairs, Sweden; the participating county councils and municipalities; and the Swedish Research Council. A specific grant was obtained from the Swedish Research Council (2020-05931). Funders had no role in the in the study design; in the collection, analysis and interpretation of the data; in the writing of the report; and in the decision to submit the paper for publication.

Competing interests: None declared.

Patient consent for publication: Not required.

Provenance and peer review: Not commissioned; externally peer reviewed.

Data availability statement: Data are from the SNAC-K project, a population-based study on aging and dementia (http://www.snac-k.se/). Access to these original data is available to the research community upon approval by the SNAC-K data management and maintenance committee. Applications for accessing these data can be submitted to Maria Wahlberg (Maria.Wahlberg@ki.se) at the Aging Research Center, Karolinska Institutet.

For peer review only

REFERENCES

1 Public Health Agency of Sweden. COVID-19 statistics in Sweden. https://www.folkhalsomyndigheten.se/smittskydd-beredskap/utbrott/aktuella-utbrott/covid-19/statistik-och-analyser/bekraftade-fall-i-sverige/ (accessed 24 Jan 2021).

2 Parohan M, Yaghoubi S, Seraji A, *et al.* Risk factors for mortality in patients with Coronavirus disease 2019 (COVID-19) infection: a systematic review and meta-analysis of observational studies. *The Aging Male* 2020;:1–9. doi:10.1080/13685538.2020.1774748

3 Tehrani S, Killander A, Åstrand P, *et al.* Risk factors for death in adult COVID-19 patients: Frailty predicts fatal outcome in older patients. *International Journal of Infectious Diseases* 2021;**102**:415–21. doi:10.1016/j.ijid.2020.10.071

4 Hawkins RB, Charles EJ, Mehaffey JH. Socio-economic status and COVID-19–related cases and fatalities. *Public Health* 2020;**189**:129–34. doi:10.1016/j.puhe.2020.09.016

5 Marengoni A, Zucchelli A, Vetrano DL, *et al.* Beyond Chronological Age: Frailty and Multimorbidity Predict In-Hospital Mortality in Patients With Coronavirus Disease 2019. *The Journals of Gerontology: Series A* 2020;:glaa291. doi:10.1093/gerona/glaa291

6 Protect yourself and others. https://www.folkhalsomyndigheten.se/globalassets/smittskydd-sjukdomar/sjukdomar/coronavirus/faktablad-pa-olika-sprak/trafikljus_engelska_what-to-do-if-you-are.pdf (accessed 25 Jan 2021).

7 Calderón-Larrañaga A, Dekhtyar S, Vetrano DL, *et al.* COVID-19: risk accumulation among biologically and socially vulnerable older populations. *Ageing Research Reviews* 2020;**63**:101149. doi:10.1016/j.arr.2020.101149

8 Courtin E, Knapp M. Social isolation, loneliness and health in old age: a scoping review. *Health Soc Care Community* 2017;**25**:799–812. doi:10.1111/hsc.12311

9 Narici M, De Vito G, Franchi M, *et al.* Impact of sedentarism due to the COVID-19 home confinement on neuromuscular, cardiovascular and metabolic health: Physiological and pathophysiological implications and recommendations for physical and nutritional countermeasures. *European Journal of Sport Science* 2020;:1–22. doi:10.1080/17461391.2020.1761076

10 Callow DD, Arnold-Nedimala NA, Jordan LS, *et al.* The Mental Health Benefits of Physical Activity in Older Adults Survive the COVID-19 Pandemic. *The American Journal of Geriatric Psychiatry* 2020;**28**:1046–57. doi:10.1016/j.jagp.2020.06.024

11 Brandén M, Aradhya S, Kolk M, *et al.* Residential context and COVID-19 mortality among adults aged 70 years and older in Stockholm: a population-based, observational study using individual-level data. *The Lancet Healthy Longevity* 2020;**1**:e80–8. doi:10.1016/S2666-7568(20)30016-7

12 Calderón-Larrañaga A, Vetrano DL, Rizzuto D, *et al.* High excess mortality in areas with young and socially vulnerable populations during the COVID-19 outbreak in Stockholm Region, Sweden. *BMJ Glob Health* 2020;**5**:e003595. doi:10.1136/bmjgh-2020-003595

For peer review only - http://bmjopen.bmj.com/site/about/guidelines.xhtml

13 Drefahl S, Wallace M, Mussino E, *et al.* A population-based cohort study of socio-demographic risk factors for COVID-19 deaths in Sweden. *Nat Commun* 2020;**11**:5097. doi:10.1038/s41467-020-18926-3

14 Gustavsson J, Beckman L. Compliance to Recommendations and Mental Health Consequences among Elderly in Sweden during the Initial Phase of the COVID-19 Pandemic—A Cross Sectional Online Survey. *IJERPH* 2020;**17**:5380. doi:10.3390/ijerph17155380

15 World Health Organization. Regional Office for Europe. Survey tool and guidance: rapid, simple, flexible behavioural insights on COVID-19: 29 July 2020. Copenhagen: 2020. https://apps.who.int/iris/handle/10665/333549

16 Hughes ME, Waite LJ, Hawkley LC, *et al.* A Short Scale for Measuring Loneliness in Large Surveys: Results From Two Population-Based Studies. *Res Aging* 2004;**26**:655–72. doi:10.1177/0164027504268574

17 Xiong J, Lipsitz O, Nasri F, *et al.* Impact of COVID-19 pandemic on mental health in the general population: A systematic review. *Journal of Affective Disorders* 2020;**277**:55–64. doi:10.1016/j.jad.2020.08.001

18 Vindegaard N, Benros ME. COVID-19 pandemic and mental health consequences: Systematic review of the current evidence. *Brain, Behavior, and Immunity* 2020;**89**:531–42. doi:10.1016/j.bbi.2020.05.048

19 van der Vegt I, Kleinberg B. Women Worry About Family, Men About the Economy: Gender Differences in Emotional Responses to COVID-19. In: Aref S, Bontcheva K, Braghieri M, *et al.*, eds. *Social Informatics*. Cham: : Springer International Publishing 2020. 397–409. doi:10.1007/978-3-030-60975-7_29

20 Schwarzbach M, Luppa M, Forstmeier S, *et al.* Social relations and depression in late life-A systematic review: Social relations and depression in late life. *Int J Geriatr Psychiatry* 2014;**29**:1–21. doi:10.1002/gps.3971

21 Jarach CM, Tettamanti M, Nobili A, *et al.* Social isolation and loneliness as related to progression and reversion of frailty in the Survey of Health Aging Retirement in Europe (SHARE). *Age and Ageing* 2020;:afaa168. doi:10.1093/ageing/afaa168

22 Evans IEM, Martyr A, Collins R, *et al.* Social Isolation and Cognitive Function in Later Life: A Systematic Review and Meta-Analysis. *JAD* 2019;**70**:S119–44. doi:10.3233/JAD-180501

23 Beridze G, Ayala A, Ribeiro O, *et al.* Are Loneliness and Social Isolation Associated with Quality of Life in Older Adults? Insights from Northern and Southern Europe. *IJERPH* 2020;**17**:8637. doi:10.3390/ijerph17228637

24 Luna E, Ruiz M, Malyutina S, *et al.* The prospective association between frequency of contact with friends and relatives and quality of life in older adults from Central and Eastern Europe. *Soc Psychiatry Psychiatr Epidemiol* Published Online First: 10 February 2020. doi:10.1007/s00127-020-01834-8

For peer review only - http://bmjopen.bmj.com/site/about/guidelines.xhtml

25 Hajek A, König H-H. Social Isolation and Loneliness of Older Adults in Times of the COVID-19 Pandemic: Can Use of Online Social Media Sites and Video Chats Assist in Mitigating Social Isolation and Loneliness? *Gerontology* 2021;**67**:121–4. doi:10.1159/000512793

26 Kahlon MK, Aksan N, Aubrey R, *et al.* Effect of Layperson-Delivered, Empathy-Focused Program of Telephone Calls on Loneliness, Depression, and Anxiety Among Adults During the COVID-19 Pandemic: A Randomized Clinical Trial. *JAMA Psychiatry* Published Online First: 23 February 2021. doi:10.1001/jamapsychiatry.2021.0113

27 da Silva VD, Tribess S, Meneguci J, *et al.* Association between frailty and the combination of physical activity level and sedentary behavior in older adults. *BMC Public Health* 2019;**19**:709. doi:10.1186/s12889-019-7062-0

28 Inoue S, Yorifuji T, Sugiyama M, *et al.* Does Habitual Physical Activity Prevent Insomnia? A Cross-Sectional and Longitudinal Study of Elderly Japanese. *Journal of Aging and Physical Activity* 2013;**21**:119–39. doi:10.1123/japa.21.2.119

29 Konsekvenser för personer 70 år och äldre av smittskyddsåtgärder mot covid19. Folkhälsomyndigheten 2021.

30 Czeisler MÉ, Marynak K, Clarke KEN, *et al.* Delay or Avoidance of Medical Care Because of COVID-19–Related Concerns — United States, June 2020. *MMWR Morb Mortal Wkly Rep* 2020;**69**:1250–7. doi:10.15585/mmwr.mm6936a4

31 Bom J, Bakx P, Schut F, *et al.* The Impact of Informal Caregiving for Older Adults on the Health of Various Types of Caregivers: A Systematic Review. *The Gerontologist* Published Online First: 3 November 2018. doi:10.1093/geront/gny137

For peer review only

Table 1. Study sample characteristics (N=1231).

**Age** mean (SD)	78.2 (8.3)
**Age** n (%)	
≤80 years	642 (52.2%)
>80 years	589 (47.8%)
**Female** n (%)	792 (64.3%)
**Education** n (%)	
High school, university or above	809 (65.7%)
Elementary school	422 (34.3%)
**Living alone** n (%)	616 (50.2%)
**COVID-19-related symptoms** n (%)	
0	801 (65.1%)
1	183 (14.9%)
2+	247 (20.0%)
**Tested for COVID-19** n (%)	
Yes, positive	9 (0.7%)
Yes, negative/unknown	53 (4.3%)
**Sought medical care** n (%)	595 (48.6%)
**Found it difficult to access healthcare services**[a] n (%)	79 (14.6%)
**Hospitalized due to confirmed or suspected COVID-19** n (%)	9 (0.7%)

[a] Subsample of those who sought medical care (n=540)

For peer review only

Table 2. Psychological burden and behavioral changes in the study sample (N=1231) during the first COVID-19 outbreak in Stockholm (March-June 2020).

**Psychological burden (n, %)**	
**Worried about getting COVID-19**	
Not at all, slightly, moderately	1032 (85.4%)
Very, extremely	176 (14.6%)
**Worried about loved one getting COVID-19**	
Not at all, slightly, moderately	907 (75.1%)
Very, extremely	300 (24.9%)
**Felt nervous/stressed**	
Never, sometimes	1112 (91.9%)
Often, very often	98 (8.1%)
**Felt lonely[a]**	
To a low extent (<5)	790 (66.6%)
To a high extent (≥5)	396 (33.4%)
**Affected by at least one item**	608 (49.8%)
**Reductions in social and physical activities (n, %)**	
**Social isolation[b]**	195 (16.3%)
**Reduced light physical activity**	352 (29.4%)
**Reduced vigorous physical activity**	326 (27.3%)
**Affected by at least one item**	676 (55.3%)
**Reductions in care use (n, %)**	
**Refrained from seeking medical care**	126 (10.3%)
**Received less home care[c,d]**	16 (8.9%)
**Affected by at least one item**	139 (11.3%)
**Sum of collateral damage dimensions (n, %)**	
**0**	273 (22.2%)
**1**	537 (43.7%)
**2**	371 (30.2%)
**3**	48 (3.9%)

[a] Based on UCLA Three-Item Loneliness Scale (range: 3-9)
[b] Reduction in physical communication without an increase in phone and/or video communication
[c] Reduction in formal care without an increase in informal care
[d] Subsample of those who received home care before the pandemic (n=180)

For peer review only - http://bmjopen.bmj.com/site/about/guidelines.xhtml

Table 3. Association (odds ratios and 95% confidence intervals) between psychological burden and behavioral changes and sociodemographic factors (N=1231) during the first COVID-19 outbreak in Stockholm (March-June 2020). Models mutually adjusted by all sociodemographic factors.

	Oldest vs youngest old	*p-value*	Female vs male	*p-value*	Low vs high education[a]	*p-value*	Living alone vs not living alone	*p-value*
**Psychological burden**								
**Worried about getting COVID-19**	0.62 [0.44,0.87]	0.006	1.41 [0.99,2.03]	0.060	0.90 [0.64,1.28]	0.575	0.91 [0.65,1.29]	0.597
**Worried about loved one getting COVID-19**	0.84 [0.64,1.11]	0.215	1.54 [1.15,2.07]	0.004	0.99 [0.75,1.33]	0.632	0.63 [0.47,0.84]	0.001
**Felt nervous/stressed**	0.73 [0.47,1.13]	0.156	2.08 [1.23,3.52]	0.006	1.04 [0.66,1.62]	0.860	1.36 [0.87,2.16]	0.175
**Felt lonely[b]**	1.08 [0.84,1.40]	0.551	1.61 [1.22,2.12]	0.001	0.83 [0.64,1.09]	0.185	1.50 [1.15,1.95]	0.003
**Affected by at least one item**	0.94 [0.74,1.19]	0.597	1.90 [1.48,2.45]	<0.001	0.97 [0.75,1.24]	0.777	1.13 [0.89,1.45]	0.320
**Reductions in social and physical activity**								
**Social isolation[c]**	1.19 [0.86,1.63]	0.295	0.64 [0.46,0.89]	0.008	1,31 [0.95, 1.82]	0.104	1.13 [0.81,1.58]	0.476
**Reduced light physical activity**	1.82 [1.40,2.37]	<0.001	1.58 [1.19,2.11]	0.002	0.94 [0.72,1.24]	0.680	1.06 [0.80,1.39]	0.692
**Reduced vigorous physical activity**	0.55 [0.42,0.72]	<0.001	1.32 [0.99,1.76]	0.056	0.79 [0.60,1.06]	0.116	0.76 [0.57,1.00]	0.05
**Affected by at least one item**	1.17 [0.93,1.49]	0.181	1.30 [1.08,1.78]	0.009	1.05 [0.82,1.34]	0.691	0.89 [0.70,1.14]	0.373
**Reductions in care use**								
**Refrained from seeking medical care**	0.98 [0.67,1.44]	0.928	1.05 [0.70,1.58]	0.801	0.94 [0.63,1.40]	0.761	1.10 [0.74,1.64]	0.637
**Received less home care[d,e]**	0.14 [0.03,0.62]	0.010	3.58 [0.63,20.4]	0.151	3.98 [1.15,13.7]	0.029	1.31 [0.30,5.63]	0.718
**Affected by at least one item**	1.06 [0.73,1.53]	0.757	1.10 [0.74,1.63]	0.643	1.11 [0.76,1.62]	0.595	1.15 [0.79,1.69]	0.468

[a] Low = elementary, high = high school, university or above
[b] Based on UCLA Three-Item Loneliness Scale (range: 3-9)
[c] Defined as reduction in physical communication without an increase in phone and/or video communication
[d] Defined as reduction in formal care without an increase in informal care
[e] Subsample of those who received home care before the pandemic (n=180)

For peer review only

Figure 1. Adherence to preventive recommendations (left) and sources of information (right) related to COVID-19 during the first outbreak in Stockholm (March-June 2020).

Figure 2. Association (odds ratios and 95% confidence intervals) between number of experienced dimensions of collateral damage and sociodemographic factors (N=1231) during the first COVID-19 outbreak in Stockholm (March-June 2020). Models mutually adjusted by all sociodemographic factors.

For peer review only

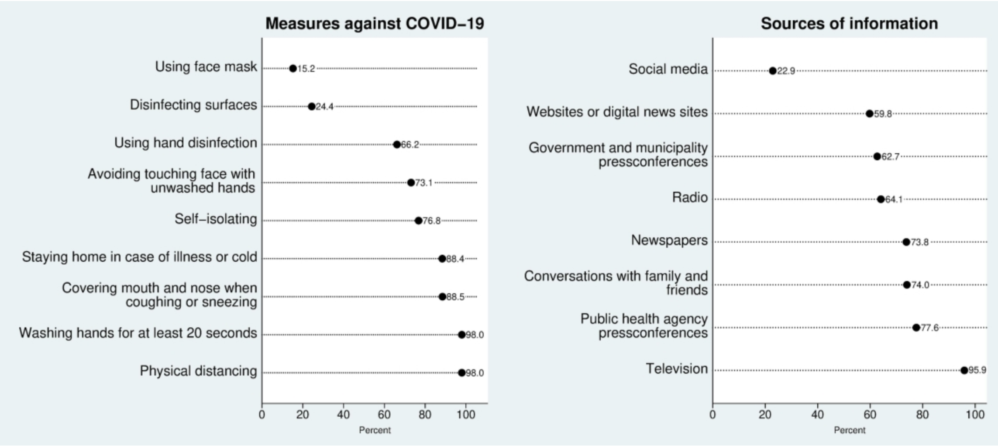

Figure 1. Adherence to preventive recommendations (left) and sources of information (right) related to COVID-19 during the first outbreak in Stockholm (March-June 2020).

228x101mm (300 x 300 DPI)

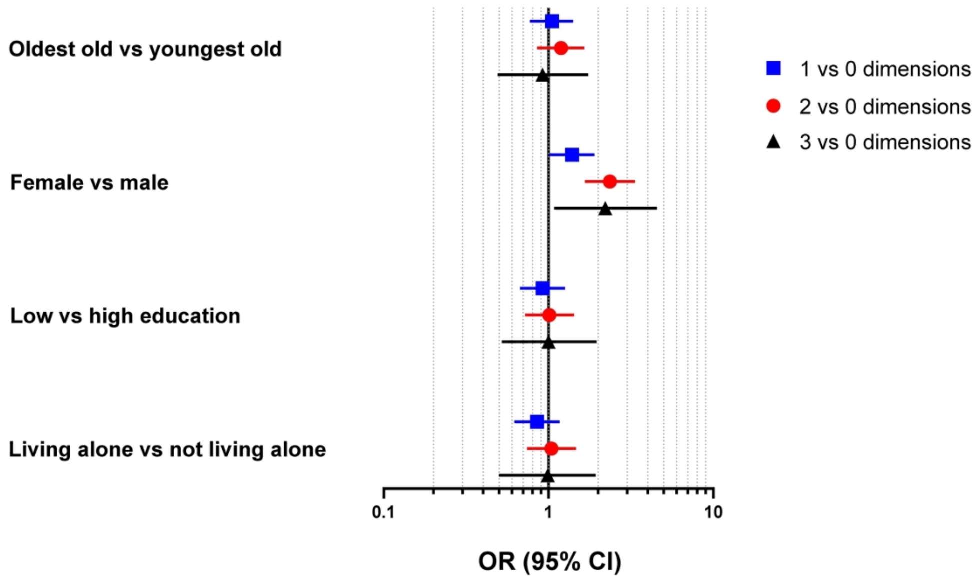

Figure 2. Association (odds ratios and 95% confidence intervals) between number of experienced dimensions of collateral damage and sociodemographic factors (N=1231) during the first COVID-19 outbreak in Stockholm (March-June 2020). Models mutually adjusted by all sociodemographic factors.

220x129mm (300 x 300 DPI)

Supplementary Table 1. Psychological distress and behavioral changes in the study sample (N=1231) by age and sex during the first COVID-19 outbreak in Stockholm (March-June 2020).

	Age		*p-value*	Sex		*p-value*
	Under 80	Over 80		Male	Female	
	n=809	n=422		n=439	n=792	
**Psychological distress**						
**Worried about getting COVID-19**			0.003			0.13
Not at all, slightly, moderately	522 (82.6%)	510 (88.5%)		378 (87.5%)	654 (84.3%)	
Very, extremely	110 (17.4%)	66 (11.5%)		54 (12.5%)	122 (15.7%)	
**Worried about loved one getting COVID-19**			0.054			0.063
Not at all, slightly, moderately	462 (72.9%)	445 (77.7%)		338 (78.2%)	569 (73.4%)	
Very, extremely	172 (27.1%)	128 (22.3%)		94 (21.8%)	206 (26.6%)	
**Felt nervous/stressed**			0.45			0.002
Never, sometimes	580 (91.3%)	532 (92.5%)		410 (95.1%)	702 (90.1%)	
Often or very often	55 (8.7%)	43 (7.5%)		21 (4.9%)	77 (9.9%)	
**Felt lonely**[a]			0.19			<0.001
To a low extent (<5)	429 (68.3%)	361 (64.7%)		319 (74.7%)	471 (62.1%)	
To a high extent (≥5)	199 (31.7%)	197 (35.3%)		108 (25.3%)	288 (37.9%)	
**Affected by at least one item**	318 (49.7%)	290 (49.8%)	0.96	171 (39.1%)	437 (55.7%)	<0.001
**Reductions in social and physical activities**						
**Social isolation**[b]	94 (14.9%)	101 (17.7%)	0.19	97 (22.8%)	255 (33.0%)	<0.001
**Reduced light physical activity**	145 (23.2%)	207 (36.2%)	<0.001	110 (26.0%)	216 (28.1%)	0.45
**Reduced vigorous physical activity**	209 (33.6%)	117 (20.5%)	<0.001	220 (50.5%)	456 (58.0%)	0.011
**Affected by at least one item**	341 (53.3%)	335 (57.6%)	0.13	83 (19.4%)	112 (14.5%)	0.025
**Reductions in care use**						
**Refrained from seeking medical care**	66 (10.3%)	60 (10.3%)	0.99	43 (9.9%)	83 (10.5%)	0.71
**Received less home care**[c,d]	4 (22.2%)	12 (7.4%)	0.036	2 (4.1%)	14 (10.7%)	0.17
**Affected by at least one item**	69 (10.8%)	70 (11.9%)	0.52	45 (10.3%)	94 (11.9%)	0.40
**Sum of collateral damage dimensions**						
**0**	147 (22.9%)	126 (21.4%)	0.40	123 (28.1%)	150 (19.0%)	<0.001
**1**	287 (44.8%)	250 (42.5%)		207 (47.3%)	330 (41.7%)	
**2**	180 (28.1%)	191 (32.5%)		95 (21.7%)	276 (34.9%)	
**3**	27 (4.2%)	21 (3.6%)		13 (3.0%)	35 (4.4%)	

[a] Based on UCLA Three-Item Loneliness Scale (range: 3-9)
[b] Reduction in physical communication without an increase in phone and/or video communication
[c] Reduction in formal care without an increase in informal care
[d] Subsample of those who received home care before the pandemic (n=180)

For peer review only

Supplementary Table 2. Psychological distress and behavioral changes in the study sample (N=1231) by education and living arrangement during the first COVID-19 outbreak in Stockholm (March-June 2020).

	Education[a]		*p-value*	Living arrangement		*p-value*
	Low	High		Living alone	Not living alone	
	n=422	n=809		n=612	n=616	
**Worried about getting COVID-19**			0.35			0.46
Not at all, slightly, moderately	360 (86.7%)	672 (84.7%)		508 (84.7%)	523 (86.2%)	
Very, extremely	55 (13.3%)	121 (15.3%)		92 (15.3%)	84 (13.8%)	
**Worried about loved one getting COVID-19**			0.66			0.004
Not at all, slightly, moderately	312 (75.9%)	595 (74.7%)		433 (71.6%)	473 (78.7%)	
Very, extremely	99 (24.1%)	201 (25.3%)		172 (28.4%)	128 (21.3%)	
**Felt nervous/stressed**			0.62			0.042
Never, sometimes	381 (91.4%)	731 (92.2%)		566 (93.6%)	545 (90.4%)	
Often or very often	36 (8.6%)	62 (7.8%)		39 (6.4%)	58 (9.6%)	
**Felt lonely[b]**			0.81			<0.001
To a low extent (<5)	269 (67.1%)	521 (66.4%)		431 (72.4%)	358 (60.8%)	
To a high extent (≥5)	132 (32.9%)	264 (33.6%)		164 (27.6%)	231 (39.2%)	
**Affected by at least one item**	213 (50.7%)	395 (49.3%)	0.63	280 (46.1%)	326 (53.3%)	0.012
**Reductions in social and physical activities**						
**Social isolation[c]**	76 (18.6%)	119 (15.0%)	0.11	95 (15.8%)	100 (16.7%)	0.67
**Reduced light physical activity**	129 (31.2%)	223 (28.4%)	0.31	156 (26.2%)	196 (32.7%)	0.013
**Reduced vigorous physical activity**	94 (22.9%)	232 (29.7%)	0.012	183 (31.0%)	142 (23.7%)	0.005
**Affected by at least one item**	239 (57.0%)	437 (54.4%)	0.38	334 (54.9%)	341 (55.7%)	0.78
**Reductions in care use**						
**Refrained from seeking medical care**	42 (10.1%)	84 (10.4%)	0.85	60 (9.9%)	66 (10.8%)	0.61
**Received less home care[d,e]**	12 (14%)	4 (4%)	0.025	3 (6.7%)	13 (9.6%)	0.55
**Affected by at least one item**	52 (12.4%)	87 (10.8%)	0.41	63 (10.3%)	76 (12.4%)	0.27
**Sum of collateral damage dimensions**						
**0**	92 (21.8%)	181 (22.4%)	0.38	141 (23.1%)	132 (21.4%)	0.046
**1**	173 (41.0%)	364 (45.1%)		285 (46.6%)	251 (40.7%)	
**2**	140 (33.2%)	231 (28.6%)		163 (26.7%)	207 (33.6%)	
**3**	17 (4.0%)	31 (3.8%)		22 (3.6%)	26 (4.2%)	

[a] Low = elementary, high = high school, university or above
[b] Based on UCLA Three-Item Loneliness Scale (range: 3-9)
[c] Reduction in physical communication without an increase in phone and/or video communication
[d] Reduction in formal care without an increase in informal care
[e] Subsample of those who received home care before the pandemic (n=180)

For peer review only - http://bmjopen.bmj.com/site/about/guidelines.xhtml

STROBE Statement—Checklist of items that should be included in reports of ***cross-sectional studies***

	Item No	Recommendation	Page No
**Title and abstract**	1	(*a*) Indicate the study's design with a commonly used term in the title or the abstract	2
		(*b*) Provide in the abstract an informative and balanced summary of what was done and what was found	2
**Introduction**			
Background/rationale	2	Explain the scientific background and rationale for the investigation being reported	4-5
Objectives	3	State specific objectives, including any prespecified hypotheses	5
**Methods**			
Study design	4	Present key elements of study design early in the paper	5
Setting	5	Describe the setting, locations, and relevant dates, including periods of recruitment, exposure, follow-up, and data collection	5-6
Participants	6	(*a*) Give the eligibility criteria, and the sources and methods of selection of participants	5
Variables	7	Clearly define all outcomes, exposures, predictors, potential confounders, and effect modifiers. Give diagnostic criteria, if applicable	5-7
Data sources/ measurement	8*	For each variable of interest, give sources of data and details of methods of assessment (measurement). Describe comparability of assessment methods if there is more than one group	*5-7*
Bias	9	Describe any efforts to address potential sources of bias	NA
Study size	10	Explain how the study size was arrived at	5
Quantitative variables	11	Explain how quantitative variables were handled in the analyses. If applicable, describe which groupings were chosen and why	6
Statistical methods	12	(*a*) Describe all statistical methods, including those used to control for confounding	7
		(*b*) Describe any methods used to examine subgroups and interactions	NA
		(*c*) Explain how missing data were addressed	7
		(*d*) If applicable, describe analytical methods taking account of sampling strategy	NA
		(*e*) Describe any sensitivity analyses	NA
**Results**			
Participants	13*	(a) Report numbers of individuals at each stage of study—eg numbers potentially eligible, examined for eligibility, confirmed eligible, included in the study, completing follow-up, and analysed	5

For peer review only - http://bmjopen.bmj.com/site/about/guidelines.xhtml

		(b) Give reasons for non-participation at each stage	5
		(c) Consider use of a flow diagram	NA
Descriptive data	14*	(a) Give characteristics of study participants (eg demographic, clinical, social) and information on exposures and potential confounders	7-8
		(b) Indicate number of participants with missing data for each variable of interest	Very high response rates; missingness can be inferred from supplementary data
Outcome data	15*	Report numbers of outcome events or summary measures	8
Main results	16	(*a*) Give unadjusted estimates and, if applicable, confounder-adjusted estimates and their precision (eg, 95% confidence interval). Make clear which confounders were adjusted for and why they were included	In all tables and figures.
		(*b*) Report category boundaries when continuous variables were categorized	In tables and their footnotes
		(*c*) If relevant, consider translating estimates of relative risk into absolute risk for a meaningful time period	NA
Other analyses	17	Report other analyses done—eg analyses of subgroups and interactions, and sensitivity analyses	NA
**Discussion**			
Key results	18	Summarise key results with reference to study objectives	9
Limitations	19	Discuss limitations of the study, taking into account sources of potential bias or imprecision. Discuss both direction and magnitude of any potential bias	11-12
Interpretation	20	Give a cautious overall interpretation of results considering objectives, limitations, multiplicity of analyses, results from similar studies, and other relevant evidence	9-11
Generalisability	21	Discuss the generalisability (external validity) of the study results	12
**Other information**			
Funding	22	Give the source of funding and the role of the funders for the present study and, if applicable, for the original study on which the present article is based	13

*Give information separately for exposed and unexposed groups.

Note: An Explanation and Elaboration article discusses each checklist item and gives methodological background and published examples of transparent reporting. The STROBE checklist is best used in conjunction with this article (freely available on the Web sites of PLoS Medicine at http://www.plosmedicine.org/, Annals of Internal Medicine at http://www.annals.org/, and Epidemiology at http://www.epidem.com/). Information on the STROBE Initiative is available at www.strobe-statement.org.

For peer review only

BMJ Open

COVID-19 collateral damage: cross-sectional study on the psychological burden and behavioral changes among older adults during the first outbreak in Stockholm, Sweden

Journal:	*BMJ Open*
Manuscript ID	bmjopen-2021-058422.R1
Article Type:	Original research
Date Submitted by the Author:	17-Nov-2021
Complete List of Authors:	Beridze, Giorgi; Karolinska Institute, Aging Research Center, Department of Neurobiology, Care Sciences and Society<br>Triolo, Federico; Karolinska Institute, Aging Research Center, Department of Neurobiology, Care Sciences and Society<br>Grande, Giulia; Karolinska Institute, Aging Research Center, Department of Neurobiology, Care Sciences and Society<br>Fratiglioni, Laura; Karolinska Institute, Aging Research Center, Department of Neurobiology, Care Sciences and Society; Stockholm Gerontology Research Center<br>Calderón-Larrañaga, Amaia; Karolinska Institute, Aging Research Center, Department of Neurobiology, Care Sciences and Society
**Primary Subject Heading**:	Epidemiology
Secondary Subject Heading:	Geriatric medicine
Keywords:	COVID-19, EPIDEMIOLOGY, GERIATRIC MEDICINE

SCHOLARONE™
Manuscripts

I, the Submitting Author has the right to grant and does grant on behalf of all authors of the Work (as defined in the below author licence), an exclusive licence and/or a non-exclusive licence for contributions from authors who are: i) UK Crown employees; ii) where BMJ has agreed a CC-BY licence shall apply, and/or iii) in accordance with the terms applicable for US Federal Government officers or employees acting as part of their official duties; on a worldwide, perpetual, irrevocable, royalty-free basis to BMJ Publishing Group Ltd ("BMJ") its licensees and where the relevant Journal is co-owned by BMJ to the co-owners of the Journal, to publish the Work in this journal and any other BMJ products and to exploit all rights, as set out in our licence.

The Submitting Author accepts and understands that any supply made under these terms is made by BMJ to the Submitting Author unless you are acting as an employee on behalf of your employer or a postgraduate student of an affiliated institution which is paying any applicable article publishing charge ("APC") for Open Access articles. Where the Submitting Author wishes to make the Work available on an Open Access basis (and intends to pay the relevant APC), the terms of reuse of such Open Access shall be governed by a Creative Commons licence – details of these licences and which Creative Commons licence will apply to this Work are set out in our licence referred to above.

Other than as permitted in any relevant BMJ Author's Self Archiving Policies, I confirm this Work has not been accepted for publication elsewhere, is not being considered for publication elsewhere and does not duplicate material already published. I confirm all authors consent to publication of this Work and authorise the granting of this licence.

For peer review only

TITLE

COVID-19 collateral damage: cross-sectional study on the psychological burden and behavioral changes among older adults during the first outbreak in Stockholm, Sweden

AUTHORS

Giorgi Beridze[1], Federico Triolo[1], Giulia Grande[1], Laura Fratiglioni[1,2], Amaia Calderón-Larrañaga[1]

AFFILIATIONS

1. Aging Research Center, Department of Neurobiology, Care Sciences and Society, Karolinska Institutet-Stockholm University, 17165 Solna, Sweden
2. Stockholm Gerontology Research Center, Stiftelsen Stockholms läns Äldrecentrum, 113 46 Stockholm, Sweden

CORRESPONDING AUTHOR

Giorgi Beridze, MD, MMSc

Tomtebodavägen 18a, Solna, Sweden 17165

giorgi.beridze@ki.se

KEYWORDS

COVID-19; collateral damage; restrictions; older adults

WORD COUNT: 2750

For peer review only

ABSTRACT

Objectives: To explore the indirect, negative effects of COVID-19 restrictions (collateral damage) on the lives and health of older adults living in central Stockholm, and to characterize the sociodemographic profile of those with the highest susceptibility to this damage.

Design: Cross-sectional study.

Setting: District of Kungsholmen in Stockholm, Sweden.

Participants: Older adults 68+ years old (n=1231) who participated in the *ad-hoc* COVID-19-related phone questionnaire administered by trained staff between May and June 2020 and had previously attended the regular follow-up assessment of the Swedish National study on Aging and Care in Kungsholmen (SNAC-K) during 2016-2019.

Primary and secondary outcome measures: Three dimensions of collateral damage: psychological burden (feelings of worry, stress and loneliness), reductions in social and physical activities, and reductions in medical and social care use since the beginning of the pandemic. Logistic regression models were used to test the association between age, sex, education and living arrangement, and the risk of collateral damage.

Results: Vast majority of participants adhered to the recommendations, with over three quarters practicing self-isolation (n=928). Half of the sample reported psychological burden, 55.3% reported reductions in social or physical activity, and 11.3% reported decreased medical or social care use. 77.8% were affected by at least one of the three collateral damage dimensions. Female sex was the strongest sociodemographic predictor of both individual and co-occurring dimensions of collateral damage.

Conclusions: COVID-19 and its restrictions during the first half of 2020 had a negative effect on the health and lives of a majority of elderly living in central Stockholm. Women were at a

For peer review only - http://bmjopen.bmj.com/site/about/guidelines.xhtml

higher risk of these negative consequences. We emphasize the need for predefined, evidence-based interventions to address these negative consequences.

STRENGTHS AND LIMITATIONS OF THIS STUDY

- This study uses a large sample of older adults from a well-characterized population-based study (SNAC-K)
- Several dimensions of the indirect, negative effects (collateral damage) of the COVID-19 pandemic are explored in this study
- As Sweden's response to COVID-19 differed from most countries, this study provides a unique opportunity of comparison with other settings
- The cross-sectional design of this study does not allow to establish temporality between the onset of the pandemic and studied outcomes
- The results of this study may not be generalizable to the entire elderly population in Sweden as participants are from an urban, affluent area in Stockholm

For peer review only

INTRODUCTION

The outbreak caused by the novel coronavirus, SARS-CoV2, was declared a global pandemic by the World Health Organization (WHO) on March 11th 2020, coinciding with the date of the first confirmed death in Sweden. Early on, it was identified that older adults were at a significantly higher risk of mortality from COVID-19. Indeed, as of January 18th 2021, 91% of deaths attributed to COVID-19 in Sweden have happened in those aged 70 and above [1]. Later on, additional prognostic factors were identified, including male sex, socioeconomic disadvantage, the presence of comorbidities and frailty [2–5].

In response to the pandemic, most countries have implemented strict measures to help curb the spread of the virus and reduce mortality. Sweden's response to COVID-19 differed from most countries by not implementing strict lockdowns and restrictions, but instead relying on high voluntary adherence to the recommendations proposed by the Public Health Agency. The general recommendations included keeping good hand hygiene, practicing social distancing and avoiding contact if having any symptoms [6]. On top of these, the specific recommendations for the elderly were to stay at home, avoid social gatherings and public transportation, but to remain physically active outdoors in a safe manner [6].

The importance of looking beyond mortality and morbidity when assessing national response strategies for COVID-19 has been raised [7]. Stay-at-home orders and lack of contact with loved ones put elderly at risk of loneliness and social isolation, which, in turn, are known to have negative effects, particularly in old age [8]. Reduced physical activity and sedentarism have detrimental effects on physical and mental health [9,10]. Additionally, due to the overburdening of healthcare services and reduced access to medical, social and informal care, new conditions may not be timely diagnosed, and existing health conditions may be exacerbated. These consequences can be considered indirect, negative effects (i.e. collateral damage) of COVID-19 restrictions.

For peer review only - http://bmjopen.bmj.com/site/about/guidelines.xhtml

While several studies have examined the distribution of COVID-19 mortality among Swedish older adults by sex, socioeconomic and household factors [11–13], little is known on the collateral damage of these restrictions. To the best of our knowledge, only one study has examined the mental health consequences of the Swedish strategy on the elderly [14], and no study has looked into other dimensions such as psychological wellbeing and/or behavioral changes. Thus, the aims of this study were to explore different dimensions of the collateral damage linked to COVID-19 during the first epidemic outbreak in an older population of central Stockholm, as well as to characterize the sociodemographic profile of those with the highest susceptibility to this damage.

METHODS

Study population

Study population consisted of 1231 older adults aged between 68 and 103, participating in the Swedish National study on Aging and Care in Kungsholmen (SNAC-K). SNAC-K is a longitudinal study including a random sample of older adults aged 60 years and above living in the Kungsholmen area of Stockholm, Sweden. All SNAC-K participants who had participated in the regular follow-up assessment during 2016-2019 were invited to participate in a telephone interview aimed at monitoring preventive behaviors and the direct and indirect health consequences of the COVID-19 pandemic. Telephone interviews were conducted between May and June 2020 (95%) by trained SNAC-K staff, following a multi-choice questionnaire that was *ad hoc* developed by the SNAC-K data collection team as well as experts in geriatric medicine, neurology and public health. The questionnaire comprised a selection of items from the original SNAC-K assessments and from the WHO Europe survey tool to monitor the public's risk perceptions, behaviors, trust and knowledge concerning the pandemic outbreak response [15]. The interview was preceded by a brief explanation whereby participants were told that all

For peer review only - http://bmjopen.bmj.com/site/about/guidelines.xhtml

questions referred specifically to the pandemic context. Exclusion criteria included living in care and nursing homes, known diagnosis of dementia and very impaired hearing. The response rate was 91.9%. Subjects who refused to participate or could not be contacted had similar age and educational attainment to those who participated, but were more likely to be male (45.4% vs 35.7%, p=0.044).

Collateral damage

In this study we examined three dimensions of collateral damage: psychological burden and two aspects related to behavioral changes, i.e. reductions in social and physical activities, and in medical and social care use. All questions explicitly asked participants about changes since the beginning of the pandemic. Psychological burden was assessed with variables related to worrying about being affected by COVID-19 (very/extremely vs. not at all/somewhat/moderately), worrying about loved ones being affected by COVID-19 (very/extremely vs. not at all/somewhat/moderately), feeling nervous and/or stressed (often/very often vs. never/sometimes), and loneliness (≥5 vs. <5 on the Three-Item Loneliness Scale [16]). The three questions on COVID19-related feelings of worry, nervousness or stress were taken directly from the first version of the WHO Europe survey tool mentioned above. The Three-Item Loneliness Scale is a short version of the R-UCLA Loneliness Scale that is part of several European aging cohorts such as the Survey of Health, Ageing and Retirement in Europe (SHARE) or the English Longitudinal Study of Ageing (ELSA). It measures indirect loneliness based on the items “companionship”, “left out”, and “isolated”, which are answered on a three point Likert scale (“often”, “some of the time”, “hardly ever or never”). The minimum of the resulting score is 3 (“not lonely”) and the maximum is 9 (“very lonely”). Changes in social and physical activities were measured by asking participants about reductions in social interactions, reductions in light physical activity (yes/no) and reductions in vigorous physical activity (yes/no). A reduction in social interactions, hereon referred to as social isolation, was

For peer review only - http://bmjopen.bmj.com/site/about/guidelines.xhtml

defined as a reduction in physical communication with family and friends without an increase in phone and/or video communication. Care-related items included refraining from seeking medical care (yes/no) and receiving reduced care at home. Reduced care at home was defined as a decrease in the use of formal home-care services without an increase in received informal care.

Preventive measures and sources of information

Participants were asked about their adherence to a list of 9 recommendations −both general and those specific to elderly−, and the most common sources of information regarding the COVID-19 pandemic.

Sociodemographics

Sociodemographic variables in the present study included age, sex, education and living arrangement. Age was dichotomized as youngest old (≤80 years old) and oldest old (>80 years old). Highest obtained education was dichotomized as low (elementary) and high (high school, university, or above). Living arrangement was dichotomized into those who lived alone and those who did not.

Statistical analysis

Characteristics of the study sample were reported as overall, as well as stratified by the four sociodemographic variables. Between-group differences were assessed via two-tailed t-tests and chi-square tests as appropriate. Binary logistic regression models were used to assess the associations between sociodemographic variables and the different collateral damage dimensions, as well as each item within these dimensions. All models were mutually adjusted for all sociodemographic variables. All statistical tests were performed in StataSE 15 (StataCorp LLC, College Station, TX, USA). Significance level (alpha) was set at 0.05 for all analyses.

For peer review only - http://bmjopen.bmj.com/site/about/guidelines.xhtml

Ethical considerations

Verbal informed consent was obtained from all participants. The study was approved by the Regional Ethics Review Board in Stockholm (dnr: 2020-02497).

Patient and public involvement

There was no direct public involvement either in the setting of the research questions or developing the study design.

RESULTS

The mean age of participants was 78.2 years, 64.3% were female, 34.3% had elementary educational attainment and 50.2% lived alone (**Table 1**). Five percent of participants (n=62) reported being tested for COVID-19, 9 of which reported testing positive. Almost half of the sample (48.3%, n=595) sought medical care during the period March-June 2020, with 79 of them finding it more difficult to access it. Nine participants reported being hospitalized for suspected or confirmed COVID-19.

The most commonly reported preventive behaviors were physical distancing of at least two meters (98.0%) and washing hands for at least 20 seconds (98.0%), followed by covering mouth and nose when coughing or sneezing (88.5%) and staying home in case of illness or cold (88.4%) (**Figure 1**). Three quarters of the sample (76.8%) reported self-isolating. The least commonly reported measure was usage of face masks (15.2%). Most participants stayed up to date on the COVID-19 pandemic using television (95.9%); over three quarters (77.6%) reported following the Public Health Agency's press-conferences. Digital sources, such as social media and online news websites were the least reported sources (22.9% and 59.8% respectively).

For peer review only

For peer review only - http://bmjopen.bmj.com/site/about/guidelines.xhtml

Half of the sample experienced certain level of psychological burden, with the most common items being loneliness (33.4%) and worrying about loved ones getting COVID-19 (24.9%) (**Table 2**). More than half (55.3%) experienced a reduction in either social or physical activities, and 11.3% either refrained from seeking medical care or received less social care at home. In total, 77.8% of participants (n=956) experienced at least one of the three dimensions of collateral damage comprising psychological burden, reductions in social and physical activities, and decreased medical and social care use. Almost half (43.7%) reported experiencing one dimension of collateral damage, while the remainder (34.1%) experienced two or more. Univariate associations between each of the four sociodemographic variables and the different collateral damage dimensions are presented in **Supplementary Tables 1** and **2.**

Women had higher odds of experiencing all items within the dimensions of psychological burden and social and physical activity reduction (**Table 3**). Within the dimension of psychological burden, the oldest old had significantly lower odds of worrying about getting COVID-19. Those who lived alone had significantly lower odds of worrying about loved ones getting COVID-19, but higher odds of loneliness. Within the dimension of social and physical activity reductions, the oldest old had significantly higher odds of reducing light physical activity, while the oldest old and those who lived alone had lower odds of decreasing vigorous activity. Within the dimension of medical and social care use reduction, those with lower education had higher odds of receiving less care at home.

Women were more likely to experience one (OR: 1.38, 95% CI: 1.01;1.90), two (OR: 2.36, 95% CI: 1.66;3.35) and all three (OR: 2.21, 95% CI: 1.08;4.55) collateral damage dimensions compared to men (**Figure 2**). No statistically significant differences were detected for age, education and living arrangement in terms of the number of experienced dimensions of collateral damage, after adjusting for the rest of sociodemographic factors.

For peer review only - http://bmjopen.bmj.com/site/about/guidelines.xhtml

DISCUSSION

In this study examining the collateral damage of COVID-19 restrictions in terms of psychological burden, reductions in social and physical activities, and decreased medical and social care use in a Swedish urban older population, we found that over three quarters of the sample was affected by at least one dimension, with women being at a considerably higher risk. We also showed that the vast majority of the study population adhered to the COVID-19 preventive measures during the first half of 2020.

Interpretation of results

Adherence to national recommendations is an important factor in mitigating the negative effects of the COVID-19 pandemic. We found that participants in our study were well-informed about the pandemic and adopted low-risk behaviors during the first wave of COVID-19 outbreak. The majority of participants followed the Public Health Agency press-conferences, likely reflecting the high social and institutional trust in Sweden [14], and adhered to the Agency's strongly recommended preventive measures.

We observe a substantial impact of the pandemic on the mental health of the elderly, with half of the sample reporting psychological burden. A fair share of the sample reported worrying about themselves and their loved ones being affected by COVID-19. Interestingly, the latter seems to be of more concern, a finding that has been replicated in another Swedish survey [14]. Loneliness and feelings of stress were also prevalent in our sample. This is in line with a large body of research showing considerable effects of the pandemic on mental health outcomes [17,18]. The burden seems to be unevenly borne by women and, to a lesser extent, by those living alone. Indeed, previous research has shown that women are at a higher risk of poor mental health [18] and worrying about family [19] during the pandemic.

For peer review only - http://bmjopen.bmj.com/site/about/guidelines.xhtml

High adherence to self-isolation recommendations, combined with a decrease in physical contact with loved ones, puts older adults at risk of social isolation. Social isolation presents a major modern-day challenge and has been associated with several negative health outcomes, such as depression [20], frailty [21], cognitive decline [22] and low quality of life [23,24]. While we did observe a reduction in frequency of physical meetings with family, friends and neighbors, this was largely met with an increase in phone and video communication with them. This is very important in the context of preventing the negative effects of loneliness and social isolation, as alternate forms of communication may buffer such effects [25,26].

Concern has been raised about reduction in physical activity as a major collateral consequence of the pandemic restrictions, as low physical activity is linked to negative cardiovascular and metabolic outcomes [9], poor mental health [10], frailty [27], and insomnia [28], among others. In spite of the Public Health Agency's recommendations for the elderly to remain physically active and spend time outdoors in a safe manner, we still found that up to a third of the sample had decreased their frequency of both light and vigorous physical activity. Furthermore, it is important to highlight that the reduction in light physical activity was more prominent among the oldest old, who, in all likelihood, were also doing less incidental physical activity, such as climbing the stairs or visiting the supermarket, due to self-isolation. They might encounter difficulties in returning to their former activity levels should the pandemic persist for a long time, which requires close monitoring from medical and social services.

We found that subjects in our sample limited their contact with the healthcare system during the first wave of the pandemic, but when seeking for help, received it in a timely and satisfactory way. This is an important finding in a context where hospital overcrowding has emerged as an important challenge in many countries. Around 10% of our sample refrained from seeking medical care altogether, which may explain, among others, the reduction in cancer diagnosis by Swedish healthcare services compared to previous years [29]. Still, the proportion of those

For peer review only - http://bmjopen.bmj.com/site/about/guidelines.xhtml

refraining was significantly lower than in the US, where a third of the population aged 65+ reported delaying or avoiding medical care during the first wave of the pandemic [30]. Subjects also decreased their use of formal care but seem to have compensated for it by an increase in received informal care. This could become a concern should the pandemic persist, since it is widely acknowledged that informal caregiving places significant economic, physical and mental burden on caregivers, who are often themselves older adults with health needs [31].

Strengths and limitations

To the best of our knowledge, this study is the first to examine the consequences of the Swedish COVID-19 strategy in a random sample of urban older adults. Further strengths include the use of an *ad hoc* questionnaire developed by a multidisciplinary team of experts, and the study sample coming from a well-characterized population-based study. Being based on data from Sweden, the study also provides a unique opportunity for comparison with other settings, as the Swedish strategy against COVID-19 differed from most countries. Certain limitations also need to be highlighted. We did not have recent pre-pandemic measures of physical and mental health. Thus, despite participants being asked to answer the questions for the period since March, the cross-sectional design does not allow us to assess temporal relationship between the onset of the pandemic and the studied outcomes. The findings from this cohort of older adults living in an affluent neighborhood of Stockholm may not be generalizable to the entire Swedish population. However, these findings could be viewed as a best-case scenario, and the identified collateral damage would be expected to be of higher magnitude in less urban and affluent parts of the country.

Conclusion

The results from this study indicate that, in addition to morbidity and mortality, COVID-19 and its related restrictions during the first half of 2020 have also resulted in changes that negatively

For peer review only

affect the health and lives of the elderly living in Central Stockholm. Furthermore, we found age-, sex-, living arrangement- and, to a much lesser extent, education-related differences in the susceptibility to these consequences, with women being at a particularly increased risk. When introducing restrictions, we emphasize the need of a predefined, evidence-based strategy to provide support, both during the pandemic and once the outbreak is overcome, to those who are most susceptible to these consequences.

For peer review only

Contributors: GB, LF, and AC-L developed the study concept and design. GB performed the data analysis and drafted the manuscript. GB, FT, GG, LF and AC-L interpreted the data, provided critical revisions and approved the final version of the manuscript for submission. All authors agree to be accountable for all aspects of the work and in ensuring that questions related to the accuracy or integrity of any part of the work are appropriately investigated and resolved.

Funding: This work was supported by the funders of the Swedish National study on Aging and Care (SNAC): the Ministry of Health and Social Affairs (grant number N/A), Sweden; the participating county councils and municipalities; and the Swedish Research Council. A specific grant was obtained from the Swedish Research Council (2020-05931). Funders had no role in the in the study design; in the collection, analysis and interpretation of the data; in the writing of the report; and in the decision to submit the paper for publication.

Competing interests: None declared.

Patient consent for publication: Not required.

Provenance and peer review: Not commissioned; externally peer reviewed.

For peer review only

Data availability statement: Data are from the SNAC-K project, a population-based study on aging and dementia (http://www.snac-k.se/). Access to these original data is available to the research community upon approval by the SNAC-K data management and maintenance committee. Applications for accessing these data can be submitted to Maria Wahlberg (Maria.Wahlberg@ki.se) at the Aging Research Center, Karolinska Institutet.

For peer review only - http://bmjopen.bmj.com/site/about/guidelines.xhtml

REFERENCES

1 Public Health Agency of Sweden. COVID-19 statistics in Sweden. https://www.folkhalsomyndigheten.se/smittskydd-beredskap/utbrott/aktuella-utbrott/covid-19/statistik-och-analyser/bekraftade-fall-i-sverige/ (accessed 24 Jan 2021).

2 Parohan M, Yaghoubi S, Seraji A, *et al.* Risk factors for mortality in patients with Coronavirus disease 2019 (COVID-19) infection: a systematic review and meta-analysis of observational studies. *The Aging Male* 2020;:1–9. doi:10.1080/13685538.2020.1774748

3 Tehrani S, Killander A, Åstrand P, *et al.* Risk factors for death in adult COVID-19 patients: Frailty predicts fatal outcome in older patients. *International Journal of Infectious Diseases* 2021;**102**:415–21. doi:10.1016/j.ijid.2020.10.071

4 Hawkins RB, Charles EJ, Mehaffey JH. Socio-economic status and COVID-19–related cases and fatalities. *Public Health* 2020;**189**:129–34. doi:10.1016/j.puhe.2020.09.016

5 Marengoni A, Zucchelli A, Vetrano DL, *et al.* Beyond Chronological Age: Frailty and Multimorbidity Predict In-Hospital Mortality in Patients With Coronavirus Disease 2019. *The Journals of Gerontology: Series A* 2020;:glaa291. doi:10.1093/gerona/glaa291

6 Protect yourself and others. https://www.folkhalsomyndigheten.se/globalassets/smittskydd-sjukdomar/sjukdomar/coronavirus/faktablad-pa-olika-sprak/trafikljus_engelska_what-to-do-if-you-are.pdf (accessed 25 Jan 2021).

7 Calderón-Larrañaga A, Dekhtyar S, Vetrano DL, *et al.* COVID-19: risk accumulation among biologically and socially vulnerable older populations. *Ageing Research Reviews* 2020;**63**:101149. doi:10.1016/j.arr.2020.101149

8 Courtin E, Knapp M. Social isolation, loneliness and health in old age: a scoping review. *Health Soc Care Community* 2017;**25**:799–812. doi:10.1111/hsc.12311

9 Narici M, De Vito G, Franchi M, *et al.* Impact of sedentarism due to the COVID-19 home confinement on neuromuscular, cardiovascular and metabolic health: Physiological and pathophysiological implications and recommendations for physical and nutritional countermeasures. *European Journal of Sport Science* 2020;:1–22. doi:10.1080/17461391.2020.1761076

10 Callow DD, Arnold-Nedimala NA, Jordan LS, *et al.* The Mental Health Benefits of Physical Activity in Older Adults Survive the COVID-19 Pandemic. *The American Journal of Geriatric Psychiatry* 2020;**28**:1046–57. doi:10.1016/j.jagp.2020.06.024

11 Brandén M, Aradhya S, Kolk M, *et al.* Residential context and COVID-19 mortality among adults aged 70 years and older in Stockholm: a population-based, observational study using individual-level data. *The Lancet Healthy Longevity* 2020;**1**:e80–8. doi:10.1016/S2666-7568(20)30016-7

12 Calderón-Larrañaga A, Vetrano DL, Rizzuto D, *et al.* High excess mortality in areas with young and socially vulnerable populations during the COVID-19 outbreak in Stockholm Region, Sweden. *BMJ Glob Health* 2020;**5**:e003595. doi:10.1136/bmjgh-2020-003595

For peer review only

13 Drefahl S, Wallace M, Mussino E, *et al.* A population-based cohort study of socio-demographic risk factors for COVID-19 deaths in Sweden. *Nat Commun* 2020;**11**:5097. doi:10.1038/s41467-020-18926-3

14 Gustavsson J, Beckman L. Compliance to Recommendations and Mental Health Consequences among Elderly in Sweden during the Initial Phase of the COVID-19 Pandemic—A Cross Sectional Online Survey. *IJERPH* 2020;**17**:5380. doi:10.3390/ijerph17155380

15 World Health Organization. Regional Office for Europe. Survey tool and guidance: rapid, simple, flexible behavioural insights on COVID-19: 29 July 2020. Copenhagen: 2020. https://apps.who.int/iris/handle/10665/333549

16 Hughes ME, Waite LJ, Hawkley LC, *et al.* A Short Scale for Measuring Loneliness in Large Surveys: Results From Two Population-Based Studies. *Res Aging* 2004;**26**:655–72. doi:10.1177/0164027504268574

17 Xiong J, Lipsitz O, Nasri F, *et al.* Impact of COVID-19 pandemic on mental health in the general population: A systematic review. *Journal of Affective Disorders* 2020;**277**:55–64. doi:10.1016/j.jad.2020.08.001

18 Vindegaard N, Benros ME. COVID-19 pandemic and mental health consequences: Systematic review of the current evidence. *Brain, Behavior, and Immunity* 2020;**89**:531–42. doi:10.1016/j.bbi.2020.05.048

19 van der Vegt I, Kleinberg B. Women Worry About Family, Men About the Economy: Gender Differences in Emotional Responses to COVID-19. In: Aref S, Bontcheva K, Braghieri M, *et al.*, eds. *Social Informatics*. Cham: : Springer International Publishing 2020. 397–409. doi:10.1007/978-3-030-60975-7_29

20 Schwarzbach M, Luppa M, Forstmeier S, *et al.* Social relations and depression in late life-A systematic review: Social relations and depression in late life. *Int J Geriatr Psychiatry* 2014;**29**:1–21. doi:10.1002/gps.3971

21 Jarach CM, Tettamanti M, Nobili A, *et al.* Social isolation and loneliness as related to progression and reversion of frailty in the Survey of Health Aging Retirement in Europe (SHARE). *Age and Ageing* 2020;:afaa168. doi:10.1093/ageing/afaa168

22 Evans IEM, Martyr A, Collins R, *et al.* Social Isolation and Cognitive Function in Later Life: A Systematic Review and Meta-Analysis. *JAD* 2019;**70**:S119–44. doi:10.3233/JAD-180501

23 Beridze G, Ayala A, Ribeiro O, *et al.* Are Loneliness and Social Isolation Associated with Quality of Life in Older Adults? Insights from Northern and Southern Europe. *IJERPH* 2020;**17**:8637. doi:10.3390/ijerph17228637

24 Luna E, Ruiz M, Malyutina S, *et al.* The prospective association between frequency of contact with friends and relatives and quality of life in older adults from Central and Eastern Europe. *Soc Psychiatry Psychiatr Epidemiol* Published Online First: 10 February 2020. doi:10.1007/s00127-020-01834-8

For peer review only

25 Hajek A, König H-H. Social Isolation and Loneliness of Older Adults in Times of the COVID-19 Pandemic: Can Use of Online Social Media Sites and Video Chats Assist in Mitigating Social Isolation and Loneliness? *Gerontology* 2021;**67**:121–4. doi:10.1159/000512793

26 Kahlon MK, Aksan N, Aubrey R, *et al.* Effect of Layperson-Delivered, Empathy-Focused Program of Telephone Calls on Loneliness, Depression, and Anxiety Among Adults During the COVID-19 Pandemic: A Randomized Clinical Trial. *JAMA Psychiatry* Published Online First: 23 February 2021. doi:10.1001/jamapsychiatry.2021.0113

27 da Silva VD, Tribess S, Meneguci J, *et al.* Association between frailty and the combination of physical activity level and sedentary behavior in older adults. *BMC Public Health* 2019;**19**:709. doi:10.1186/s12889-019-7062-0

28 Inoue S, Yorifuji T, Sugiyama M, *et al.* Does Habitual Physical Activity Prevent Insomnia? A Cross-Sectional and Longitudinal Study of Elderly Japanese. *Journal of Aging and Physical Activity* 2013;**21**:119–39. doi:10.1123/japa.21.2.119

29 Konsekvenser för personer 70 år och äldre av smittskyddsåtgärder mot covid19. Folkhälsomyndigheten 2021.

30 Czeisler MÉ, Marynak K, Clarke KEN, *et al.* Delay or Avoidance of Medical Care Because of COVID-19–Related Concerns — United States, June 2020. *MMWR Morb Mortal Wkly Rep* 2020;**69**:1250–7. doi:10.15585/mmwr.mm6936a4

31 Bom J, Bakx P, Schut F, *et al.* The Impact of Informal Caregiving for Older Adults on the Health of Various Types of Caregivers: A Systematic Review. *The Gerontologist* Published Online First: 3 November 2018. doi:10.1093/geront/gny137

For peer review only

Table 1. Study sample characteristics (N=1231).

**Age** mean (SD)	78.2 (8.3)
**Age** n (%)	
≤80 years	642 (52.2%)
>80 years	589 (47.8%)
**Female** n (%)	792 (64.3%)
**Education** n (%)	
High school, university or above	809 (65.7%)
Elementary school	422 (34.3%)
**Living alone** n (%)	616 (50.2%)
**COVID-19-related symptoms** n (%)	
0	801 (65.1%)
1	183 (14.9%)
2+	247 (20.0%)
**Tested for COVID-19** n (%)	
Yes, positive	9 (0.7%)
Yes, negative/unknown	53 (4.3%)
**Sought medical care** n (%)	595 (48.6%)
**Found it difficult to access healthcare services**[a] n (%)	79 (14.6%)
**Hospitalized due to confirmed or suspected COVID-19** n (%)	9 (0.7%)

[a] Subsample of those who sought medical care (n=540)

For peer review only - http://bmjopen.bmj.com/site/about/guidelines.xhtml

Table 2. Psychological burden and behavioral changes in the study sample (N=1231) during the first COVID-19 outbreak in Stockholm (March-June 2020).

**Psychological burden (n, %)**	
**Worried about getting COVID-19**	
Not at all, slightly, moderately	1032 (85.4%)
Very, extremely	176 (14.6%)
**Worried about loved one getting COVID-19**	
Not at all, slightly, moderately	907 (75.1%)
Very, extremely	300 (24.9%)
**Felt nervous/stressed**	
Never, sometimes	1112 (91.9%)
Often, very often	98 (8.1%)
**Felt lonely**[a]	
To a low extent (<5)	790 (66.6%)
To a high extent (≥5)	396 (33.4%)
**Affected by at least one item**	608 (49.8%)
**Reductions in social and physical activities (n, %)**	
**Social isolation**[b]	195 (16.3%)
**Reduced light physical activity**	352 (29.4%)
**Reduced vigorous physical activity**	326 (27.3%)
**Affected by at least one item**	676 (55.3%)
**Reductions in care use (n, %)**	
**Refrained from seeking medical care**	126 (10.3%)
**Received less home care**[c,d]	16 (8.9%)
**Affected by at least one item**	139 (11.3%)
**Sum of collateral damage dimensions (n, %)**	
**0**	273 (22.2%)
**1**	537 (43.7%)
**2**	371 (30.2%)
**3**	48 (3.9%)

[a] Based on UCLA Three-Item Loneliness Scale (range: 3-9)
[b] Reduction in physical communication without an increase in phone and/or video communication
[c] Reduction in formal care without an increase in informal care
[d] Subsample of those who received home care before the pandemic (n=180)

For peer review only - http://bmjopen.bmj.com/site/about/guidelines.xhtml

Table 3. Association (odds ratios and 95% confidence intervals) between psychological burden and behavioral changes and sociodemographic factors (N=1231) during the first COVID-19 outbreak in Stockholm (March-June 2020). Models mutually adjusted by all sociodemographic factors.

	Oldest vs youngest old	*p-value*	Female vs male	*p-value*	Low vs high education[a]	*p-value*	Living alone vs not living alone	*p-value*
**Psychological burden**								
Worried about getting COVID-19	0.62 [0.44,0.87]	0.006	1.41 [0.99,2.03]	0.060	0.90 [0.64,1.28]	0.575	0.91 [0.65,1.29]	0.597
Worried about loved one getting COVID-19	0.84 [0.64,1.11]	0.215	1.54 [1.15,2.07]	0.004	0.99 [0.75,1.33]	0.632	0.63 [0.47,0.84]	0.001
Felt nervous/stressed	0.73 [0.47,1.13]	0.156	2.08 [1.23,3.52]	0.006	1.04 [0.66,1.62]	0.860	1.36 [0.87,2.16]	0.175
Felt lonely[b]	1.08 [0.84,1.40]	0.551	1.61 [1.22,2.12]	0.001	0.83 [0.64,1.09]	0.185	1.50 [1.15,1.95]	0.003
Affected by at least one item	0.94 [0.74,1.19]	0.597	1.90 [1.48,2.45]	<0.001	0.97 [0.75,1.24]	0.777	1.13 [0.89,1.45]	0.320
**Reductions in social and physical activity**								
Social isolation[c]	1.19 [0.86,1.63]	0.295	0.64 [0.46,0.89]	0.008	1,31 [0.95, 1.82]	0.104	1.13 [0.81,1.58]	0.476
Reduced light physical activity	1.82 [1.40,2.37]	<0.001	1.58 [1.19,2.11]	0.002	0.94 [0.72,1.24]	0.680	1.06 [0.80,1.39]	0.692
Reduced vigorous physical activity	0.55 [0.42,0.72]	<0.001	1.32 [0.99,1.76]	0.056	0.79 [0.60,1.06]	0.116	0.76 [0.57,1.00]	0.05
Affected by at least one item	1.17 [0.93,1.49]	0.181	1.30 [1.08,1.78]	0.009	1.05 [0.82,1.34]	0.691	0.89 [0.70,1.14]	0.373
**Reductions in care use**								
Refrained from seeking medical care	0.98 [0.67,1.44]	0.928	1.05 [0.70,1.58]	0.801	0.94 [0.63,1.40]	0.761	1.10 [0.74,1.64]	0.637
Received less home care[d,e]	0.14 [0.03,0.62]	0.010	3.58 [0.63,20.4]	0.151	3.98 [1.15,13.7]	0.029	1.31 [0.30,5.63]	0.718
Affected by at least one item	1.06 [0.73,1.53]	0.757	1.10 [0.74,1.63]	0.643	1.11 [0.76,1.62]	0.595	1.15 [0.79,1.69]	0.468

[a] Low = elementary, high = high school, university or above
[b] Based on UCLA Three-Item Loneliness Scale (range: 3-9)
[c] Defined as reduction in physical communication without an increase in phone and/or video communication
[d] Defined as reduction in formal care without an increase in informal care
[e] Subsample of those who received home care before the pandemic (n=180)

For peer review only

For peer review only - http://bmjopen.bmj.com/site/about/guidelines.xhtml

Figure 1. Adherence to preventive recommendations (left) and sources of information (right) related to COVID-19 during the first outbreak in Stockholm (March-June 2020).

Figure 2. Association (odds ratios and 95% confidence intervals) between number of experienced dimensions of collateral damage and sociodemographic factors (N=1231) during the first COVID-19 outbreak in Stockholm (March-June 2020). Models mutually adjusted by all sociodemographic factors.

For peer review only

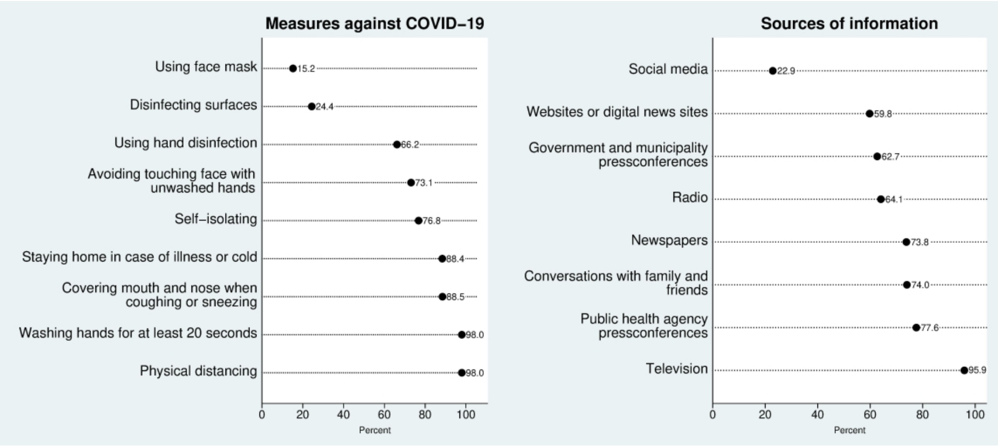

Figure 1. Adherence to preventive recommendations (left) and sources of information (right) related to COVID-19 during the first outbreak in Stockholm (March-June 2020).

228x101mm (600 x 600 DPI)

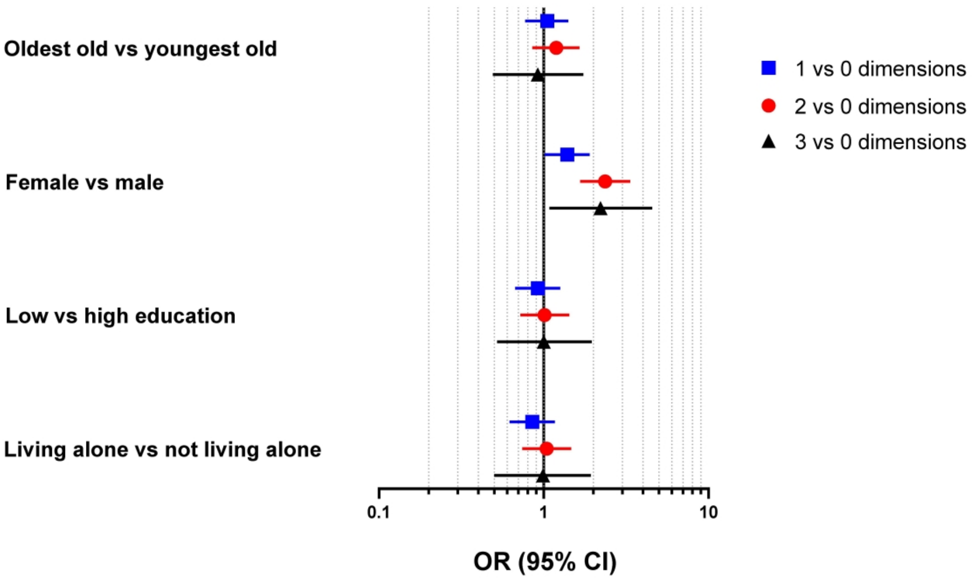

Figure 2. Association (odds ratios and 95% confidence intervals) between number of experienced dimensions of collateral damage and sociodemographic factors (N=1231) during the first COVID-19 outbreak in Stockholm (March-June 2020). Models mutually adjusted by all sociodemographic factors.

220x129mm (600 x 600 DPI)

Supplementary Table 1. Psychological distress and behavioral changes in the study sample (N=1231) by age and sex during the first COVID-19 outbreak in Stockholm (March-June 2020).

	Age		*p-value*	Sex		*p-value*
	Under 80	Over 80		Male	Female	
	n=809	n=422		n=439	n=792	
**Psychological distress**						
**Worried about getting COVID-19**			0.003			0.13
Not at all, slightly, moderately	522 (82.6%)	510 (88.5%)		378 (87.5%)	654 (84.3%)	
Very, extremely	110 (17.4%)	66 (11.5%)		54 (12.5%)	122 (15.7%)	
**Worried about loved one getting COVID-19**			0.054			0.063
Not at all, slightly, moderately	462 (72.9%)	445 (77.7%)		338 (78.2%)	569 (73.4%)	
Very, extremely	172 (27.1%)	128 (22.3%)		94 (21.8%)	206 (26.6%)	
**Felt nervous/stressed**			0.45			0.002
Never, sometimes	580 (91.3%)	532 (92.5%)		410 (95.1%)	702 (90.1%)	
Often or very often	55 (8.7%)	43 (7.5%)		21 (4.9%)	77 (9.9%)	
**Felt lonely[a]**			0.19			<0.001
To a low extent (<5)	429 (68.3%)	361 (64.7%)		319 (74.7%)	471 (62.1%)	
To a high extent (≥5)	199 (31.7%)	197 (35.3%)		108 (25.3%)	288 (37.9%)	
**Affected by at least one item**	318 (49.7%)	290 (49.8%)	0.96	171 (39.1%)	437 (55.7%)	<0.001
**Reductions in social and physical activities**						
**Social isolation[b]**	94 (14.9%)	101 (17.7%)	0.19	97 (22.8%)	255 (33.0%)	<0.001
**Reduced light physical activity**	145 (23.2%)	207 (36.2%)	<0.001	110 (26.0%)	216 (28.1%)	0.45
**Reduced vigorous physical activity**	209 (33.6%)	117 (20.5%)	<0.001	220 (50.5%)	456 (58.0%)	0.011
**Affected by at least one item**	341 (53.3%)	335 (57.6%)	0.13	83 (19.4%)	112 (14.5%)	0.025
**Reductions in care use**						
**Refrained from seeking medical care**	66 (10.3%)	60 (10.3%)	0.99	43 (9.9%)	83 (10.5%)	0.71
**Received less home care[c,d]**	4 (22.2%)	12 (7.4%)	0.036	2 (4.1%)	14 (10.7%)	0.17
**Affected by at least one item**	69 (10.8%)	70 (11.9%)	0.52	45 (10.3%)	94 (11.9%)	0.40
**Sum of collateral damage dimensions**						
**0**	147 (22.9%)	126 (21.4%)	0.40	123 (28.1%)	150 (19.0%)	<0.001
**1**	287 (44.8%)	250 (42.5%)		207 (47.3%)	330 (41.7%)	
**2**	180 (28.1%)	191 (32.5%)		95 (21.7%)	276 (34.9%)	
**3**	27 (4.2%)	21 (3.6%)		13 (3.0%)	35 (4.4%)	

[a] Based on UCLA Three-Item Loneliness Scale (range: 3-9)
[b] Reduction in physical communication without an increase in phone and/or video communication
[c] Reduction in formal care without an increase in informal care
[d] Subsample of those who received home care before the pandemic (n=180)

For peer review only

Supplementary Table 2. Psychological distress and behavioral changes in the study sample (N=1231) by education and living arrangement during the first COVID-19 outbreak in Stockholm (March-June 2020).

	Education[a]		*p-value*	Living arrangement		*p-value*
	Low	High		Living alone	Not living alone	
	n=422	n=809		n=612	n=616	
**Worried about getting COVID-19**			0.35			0.46
Not at all, slightly, moderately	360 (86.7%)	672 (84.7%)		508 (84.7%)	523 (86.2%)	
Very, extremely	55 (13.3%)	121 (15.3%)		92 (15.3%)	84 (13.8%)	
**Worried about loved one getting COVID-19**			0.66			0.004
Not at all, slightly, moderately	312 (75.9%)	595 (74.7%)		433 (71.6%)	473 (78.7%)	
Very, extremely	99 (24.1%)	201 (25.3%)		172 (28.4%)	128 (21.3%)	
**Felt nervous/stressed**			0.62			0.042
Never, sometimes	381 (91.4%)	731 (92.2%)		566 (93.6%)	545 (90.4%)	
Often or very often	36 (8.6%)	62 (7.8%)		39 (6.4%)	58 (9.6%)	
**Felt lonely[b]**			0.81			<0.001
To a low extent (<5)	269 (67.1%)	521 (66.4%)		431 (72.4%)	358 (60.8%)	
To a high extent (≥5)	132 (32.9%)	264 (33.6%)		164 (27.6%)	231 (39.2%)	
**Affected by at least one item**	213 (50.7%)	395 (49.3%)	0.63	280 (46.1%)	326 (53.3%)	0.012
**Reductions in social and physical activities**						
**Social isolation[c]**	76 (18.6%)	119 (15.0%)	0.11	95 (15.8%)	100 (16.7%)	0.67
**Reduced light physical activity**	129 (31.2%)	223 (28.4%)	0.31	156 (26.2%)	196 (32.7%)	0.013
**Reduced vigorous physical activity**	94 (22.9%)	232 (29.7%)	0.012	183 (31.0%)	142 (23.7%)	0.005
**Affected by at least one item**	239 (57.0%)	437 (54.4%)	0.38	334 (54.9%)	341 (55.7%)	0.78
**Reductions in care use**						
**Refrained from seeking medical care**	42 (10.1%)	84 (10.4%)	0.85	60 (9.9%)	66 (10.8%)	0.61
**Received less home care[d,e]**	12 (14%)	4 (4%)	0.025	3 (6.7%)	13 (9.6%)	0.55
**Affected by at least one item**	52 (12.4%)	87 (10.8%)	0.41	63 (10.3%)	76 (12.4%)	0.27
**Sum of collateral damage dimensions**						
**0**	92 (21.8%)	181 (22.4%)	0.38	141 (23.1%)	132 (21.4%)	0.046
**1**	173 (41.0%)	364 (45.1%)		285 (46.6%)	251 (40.7%)	
**2**	140 (33.2%)	231 (28.6%)		163 (26.7%)	207 (33.6%)	
**3**	17 (4.0%)	31 (3.8%)		22 (3.6%)	26 (4.2%)	

[a] Low = elementary, high = high school, university or above
[b] Based on UCLA Three-Item Loneliness Scale (range: 3-9)
[c] Reduction in physical communication without an increase in phone and/or video communication
[d] Reduction in formal care without an increase in informal care
[e] Subsample of those who received home care before the pandemic (n=180)

For peer review only - http://bmjopen.bmj.com/site/about/guidelines.xhtml

STROBE Statement—Checklist of items that should be included in reports of ***cross-sectional studies***

	Item No	Recommendation	Page No
**Title and abstract**	1	(*a*) Indicate the study's design with a commonly used term in the title or the abstract	2
		(*b*) Provide in the abstract an informative and balanced summary of what was done and what was found	2
**Introduction**			
Background/rationale	2	Explain the scientific background and rationale for the investigation being reported	4-5
Objectives	3	State specific objectives, including any prespecified hypotheses	5
**Methods**			
Study design	4	Present key elements of study design early in the paper	5
Setting	5	Describe the setting, locations, and relevant dates, including periods of recruitment, exposure, follow-up, and data collection	5-6
Participants	6	(*a*) Give the eligibility criteria, and the sources and methods of selection of participants	5
Variables	7	Clearly define all outcomes, exposures, predictors, potential confounders, and effect modifiers. Give diagnostic criteria, if applicable	5-7
Data sources/ measurement	8*	For each variable of interest, give sources of data and details of methods of assessment (measurement). Describe comparability of assessment methods if there is more than one group	*5-7*
Bias	9	Describe any efforts to address potential sources of bias	NA
Study size	10	Explain how the study size was arrived at	5
Quantitative variables	11	Explain how quantitative variables were handled in the analyses. If applicable, describe which groupings were chosen and why	6
Statistical methods	12	(*a*) Describe all statistical methods, including those used to control for confounding	7
		(*b*) Describe any methods used to examine subgroups and interactions	NA
		(*c*) Explain how missing data were addressed	7
		(*d*) If applicable, describe analytical methods taking account of sampling strategy	NA
		(*e*) Describe any sensitivity analyses	NA
**Results**			
Participants	13*	(a) Report numbers of individuals at each stage of study—eg numbers potentially eligible, examined for eligibility, confirmed eligible, included in the study, completing follow-up, and analysed	5

For peer review only

		(b) Give reasons for non-participation at each stage	5
		(c) Consider use of a flow diagram	NA
Descriptive data	14*	(a) Give characteristics of study participants (eg demographic, clinical, social) and information on exposures and potential confounders	7-8
		(b) Indicate number of participants with missing data for each variable of interest	Very high response rates; missingness can be inferred from supplementary data
Outcome data	15*	Report numbers of outcome events or summary measures	8
Main results	16	(*a*) Give unadjusted estimates and, if applicable, confounder-adjusted estimates and their precision (eg, 95% confidence interval). Make clear which confounders were adjusted for and why they were included	In all tables and figures.
		(*b*) Report category boundaries when continuous variables were categorized	In tables and their footnotes
		(*c*) If relevant, consider translating estimates of relative risk into absolute risk for a meaningful time period	NA
Other analyses	17	Report other analyses done—eg analyses of subgroups and interactions, and sensitivity analyses	NA
**Discussion**			
Key results	18	Summarise key results with reference to study objectives	9
Limitations	19	Discuss limitations of the study, taking into account sources of potential bias or imprecision. Discuss both direction and magnitude of any potential bias	11-12
Interpretation	20	Give a cautious overall interpretation of results considering objectives, limitations, multiplicity of analyses, results from similar studies, and other relevant evidence	9-11
Generalisability	21	Discuss the generalisability (external validity) of the study results	12
**Other information**			
Funding	22	Give the source of funding and the role of the funders for the present study and, if applicable, for the original study on which the present article is based	13

*Give information separately for exposed and unexposed groups.

Note: An Explanation and Elaboration article discusses each checklist item and gives methodological background and published examples of transparent reporting. The STROBE checklist is best used in conjunction with this article (freely available on the Web sites of PLoS Medicine at http://www.plosmedicine.org/, Annals of Internal Medicine at http://www.annals.org/, and Epidemiology at http://www.epidem.com/). Information on the STROBE Initiative is available at www.strobe-statement.org.

For peer review only - http://bmjopen.bmj.com/site/about/guidelines.xhtml
