# Supplementary material for: COVID-19 collateral damage—psychological burden and behavioural changes among older adults during the first outbreak in Stockholm, Sweden: a cross-sectional study
Source: BMJ Open. 2022 Jan 7;12(1):e058422. doi: 10.1136/bmjopen-2021-058422 (PMC8743841; doi:10.1136/bmjopen-2021-058422)
Supplement: Reviewer comments [file bmjopen-2021-058422.reviewer_comments.pdf]

PEER REVIEW HISTORY

BMJ Open publishes all reviews undertaken for accepted manuscripts. Reviewers are asked to complete a checklist review form (**http://bmjopen.bmj.com/site/about/resources/checklist.pdf**) and are provided with free text boxes to elaborate on their assessment. These free text comments are reproduced below.

ARTICLE DETAILS

**TITLE (PROVISIONAL)**	COVID-19 collateral damage: cross-sectional study on the psychological burden and behavioral changes among older adults during the first outbreak in Stockholm, Sweden
**AUTHORS**	Beridze, Giorgi; Triolo, Federico; Grande, Giulia; Fratiglioni, Laura; Calderón-Larrañaga, Amaia

VERSION 1 – REVIEW

**REVIEWER**	José Mira<br>Miguel Hernandez University of Elche, Health Psychology
**REVIEW RETURNED**	01-Nov-2021

**GENERAL COMMENTS**	Authors have answered one-by-one all comment and suggestion. When adequate they have introduced additional details in the main text. In my opinion, all changes are adequate. All of the identified gaps have been addressed in a right manner.
